# Discovery of a non-nucleoside inhibitor that binds to a novel site in the palm domain of the respiratory syncytial virus RNA-dependent RNA polymerase

**DOI:** 10.1128/jvi.00178-25

**Published:** 2025-06-02

**Authors:** Jay H. Kalin, Yanting Yin, Minh T. Tran, Madison Piassek, Amy Fung, Sandrine Grosse, Edgar Jacoby, Anusarka Bhaumik, Suraj Adhikary, Robyn Miller, Cynthia Lemmens, Ferdinand H. Lutter, Serge Pieters, Ludwig Cooymans, Geert Rombouts, Daniel Oehlrich, Sonia Tomaso, Kate Lozada, Miguel Osorio Garcia, Brandon Anson, Suzanne De Bruyn, Constance Smith-Monroy, Jean-Marc Neefs, Nádia Conceição-Neto, Bart Stoops, Herman van Vlijmen, Aaron Patrick, Xiaodi Yu, Victoria Wong, Daniel Krosky, Pravien Abeywickrema, Stephen Mason, Zhinan Jin, Tim H. M. Jonckers, Sujata Sharma

**Affiliations:** 1 Janssen Research & Development, LLC, a Johnson & Johnson Company, Spring House, Pennsylvania, USA; 2 Janssen Pharmaceutica N.V., Beerse, Belgium; 3 Janssen Research & Development, LLC, a Johnson & Johnson Company, Brisbane, California, USA; Loyola University Chicago - Health Sciences Campus, Maywood, Illinois, USA

**Keywords:** respiratory syncytial virus, RSV polymerase inhibitor, palm domain, NNI, cryogenic electron microscopy, high-throughput screen

## Abstract

Respiratory syncytial virus (RSV) is a major cause of severe respiratory tract infections in infants, young children, and the elderly. We report herein the discovery and characterization of a novel RSV polymerase (RSVpol) non-nucleoside inhibitor (NNI) chemotype that binds to a previously undescribed, highly conserved site in the palm domain of the L protein. Consistent with the observed mode of inhibition, cryogenic electron microscopy (cryo-EM) revealed the site to be adjacent to the nucleotide binding site. Minireplicon assays confirmed on-target activity against RSVpol, and cell-based antiviral assays showed that the lead compound effectively inhibited viral mRNA transcription and replication in clinically relevant A and B strains. Together, our data provides valuable insights into the molecular basis of inhibition for a novel mechanism of action and paves the way for structure-based design to deliver effective therapeutics against RSV.

**IMPORTANCE:**

Respiratory syncytial virus (RSV) is a negative-sense, single-stranded RNA virus belonging to the family *Pneumoviridae* of the order *Mononegavirales*. Currently, monoclonal antibody treatments are only approved for infants, and vaccines are reserved for pregnant women and adults aged 60 years and older. Prophylaxis is also limited to the pediatric patient population, and there are currently no direct antiviral therapies for post-exposure treatment. Viral polymerases are considered well-validated drug targets due to their critical role in transcription and genome replication. Herein, we disclose the discovery of a spiro-indolinone series as polymerase inhibitors and describe the preliminary structure-activity relationship (SAR). A cryogenic electron microscopy (cryo-EM) structure obtained with an optimized lead revealed a novel binding site located in the palm domain, which will enable future structure-based drug design efforts. Novel RSV antivirals could be beneficial both as therapeutics following diagnosis and as a prophylactic in patients less likely to respond to vaccines.

## INTRODUCTION

Respiratory syncytial virus (RSV) is a major cause of acute lower respiratory infections in infants, the elderly, immunocompromised individuals, and patients with underlying comorbidities, contributing to substantial morbidity and mortality worldwide (1–5). Recent progress in vaccine development against RSV has been encouraging. In 2023, Arexvy, an RSV prefusion F protein-based vaccine, was authorized for use in adults aged 60 and over in the USA, marking the first such approval for an RSV vaccine (6). This was followed by the approval of Abrysvo, a bivalent RSV prefusion F protein-based vaccine, for the same age group and also for pregnant women to safeguard infants against severe RSV diseases (7, 8). Shortly thereafter, in 2024, mResvia, the first mRNA-based vaccine targeting the prefusion F protein, was approved for use in adults aged 60 and older (9). Since 1998, palivizumab, a monoclonal antibody that targets RSV’s F protein, has been administered as a prophylactic treatment for high-risk infants (10, 11). In 2023, Nirsevimab, a novel anti-F protein monoclonal antibody, was approved for infant prophylaxis, offering a lower dosage and extended protection compared to Palivizumab (12, 13). Although these vaccines and monoclonal antibodies offer protection to specific groups, there remains a need for effective pre-exposure prophylaxis and post-exposure treatments for RSV infection, particularly for patients who cannot use these preventative modalities. The development of small-molecule therapies against RSV infection has been challenging, and current ongoing efforts mainly target the fusion protein, N protein, and the viral RNA-dependent RNA polymerase (RdRp) (14–25).

Viral RdRp is a promising antiviral drug target as evidenced by approved nucleoside and non-nucleoside (NNI) antivirals targeting the polymerase of hepatitis C virus (HCV) (26). RSV polymerase (RSVpol) is a protein complex consisting of the large protein (L) harboring the RdRp, polyribonucleotidyl transferase (PRNTase) or capping, and methyltransferase (MTase) activities, along with four phosphoproteins (P) (27). RSV P protein acts as a crucial component to control the efficiency of L, interacting with nucleocapsid (N), and guiding the newly synthesized RNA into the L active site during transcription and replication (28–30).

A cryogenic electron microscopy (cryo-EM) structure of the RSV L+P complex revealed the binding stoichiometry to be four P to one L in an asymmetric tentacular arrangement (27, 31). In the structure, the RdRp and capping domains of RSV L are visible, with the C-terminal connector and MTase domains missing due to their dynamic nature. The RdRp domain contains the classic polymerase subdomains, fingers, palm, and thumb. The RSV RdRp is a nucleotidyl-transferase responsible for *de novo* RNA synthesis for both transcription and replication of the viral RNA genome (32). It catalyzes phosphodiester bond formation from two nucleotide triphosphates (*de novo*) or from one nucleotide and a primer (elongation) and releases one pyrophosphate (PP_i_) for each nucleotide addition (33). *In vitro* RNA synthesis assays using recombinant RSV L+P complex and synthesized RNA templates have been developed to capture RdRp *de novo* and primer-dependent activities (32, 34–38). The RSV capping and MTase domains contribute to the formation of capped and methylated mRNAs (39). Recent cryo-EM structures of the RSV L+P in complex with NNIs, JNJ-8003, **S1**, and MRK-1 (Fig. S1) uncovered an induced fit inhibitor binding pocket on the capping domain (36, 40, 41). Interestingly, the binding of these inhibitors to the capping domain inhibited nucleotide addition activity of RdRp at *de novo* and early elongation steps, suggesting an interplay between the capping and the RdRp domains. Similarly, inhibitors that are hypothesized to bind in the connector domain based on their resistance profiles can inhibit both *de novo* and early elongation (e.g., JNJ-7184, AZ-27) or early elongation (e.g., AVG) (24, 42–44). However, these compounds cannot disrupt committed replication complexes and therefore do not inhibit late elongation (24). The allosteric inhibitory effects of these capping and connector domain binding inhibitors on RdRp activity suggest the RSV L+P complex has multiple druggable sites for RdRp inhibition (28–30). To search for novel RSVpol inhibitor chemotypes, a high-throughput screen (HTS) was conducted utilizing recombinant RSV L+P polymerase complex in a *de novo* RNA synthesis assay, which led to the discovery of an inhibitor targeting a previously uncharacterized binding pocket in the palm domain of the L protein. The inhibitor potency, selectivity, and binding mode were characterized, and its cryo-EM structure was elucidated with specific features. Additionally, the mechanism of action of this inhibitor was investigated, and structure-activity relationship (SAR) strategies were employed to enhance its structural and functional attributes for potential therapeutic applications.

## RESULTS

### Design and characterization of the truncated RSV L+P construct

To identify novel RSVpol inhibitor chemotypes, we conducted an HTS campaign using a luminescence-coupled enzyme assay to detect PP_i_ production (34, 38). PP_i_ liberated during RdRp-catalyzed nucleotide incorporation is converted to ATP by ATP sulfurylase, which then serves as a substrate for luciferase (Fig. S2A). In this assay, adenosine-5′-(α-thio)-triphosphate (ATPαS) is used in place of ATP as a substrate for the polymerase since it is not an efficient luciferase substrate.

**Fig 1:**
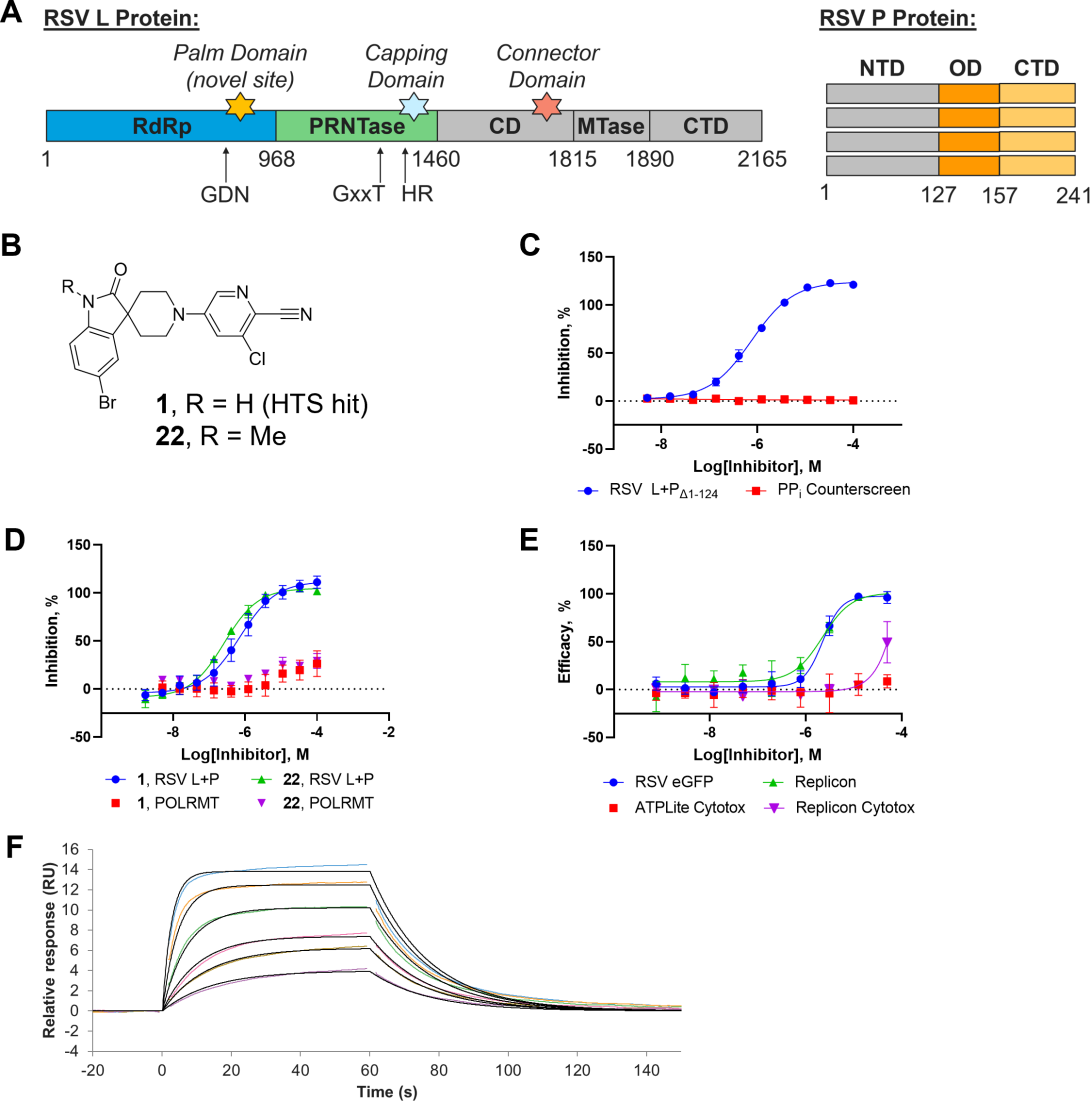
Optimized high-throughput screening hit inhibits RSV RdRp and exhibits cell-based antiviral activity. (**A**) Domain architecture of RSV L and P protein subunits. Stars indicate known inhibitor binding sites (capping domain: JNJ-8003, MRK-1, **S1**; connector domain: JNJ-7184, AZ-27, AVG), colored domains indicate structured regions observed by cryo-EM (RdRp: polymerase, PRNTase: capping, CD: connector, MTase: methyltransferase, CTD: C-terminal domain, NTD: N-terminal domain, OD: oligomerization domain). (**B**) Structure of hit and optimized cell-active lead. (**C**) Inhibition of RSV L+P screening construct and coupled enzyme system by **1**. RSV L+P_Δ1-124_ IC_50_ = 0.61 µM, PP_i_ counterscreen IC_50_ > 100 µM. RSV L+P_Δ1-124_ assay: 20 nM RSV L+P_Δ1-124_, 10 µM ATP/GTP/CTP, 100 µM ATPαS, 5 µM TrC-25. Counterscreen: 1.5 µM PP_i_, 1 U/µL ATP sulfurylase, 2 nM luciferase, 5 µM adenosine phosphosulfate (APS), 300 µM luciferin, 1 mM coenzyme A. (**D**) Biochemical activity of **1** and **22** against full-length RSV L+P and human mitochondrial RNA polymerase (POLRMT). RSV L+P IC_50_ = 0.67 µM (**1**), 0.27 µM (**22**), POLRMT IC_50_ > 100 µM (**1**, **22**). POLRMT counterscreen: 8 nM POLRMT, 4.5 µM ATP, 0.85 µM GTP, 0.1 µM CTP, 0.60 µM UTP, 0.1 µM RNA primer/ssDNA-FAM template/ssDNA-BHQ quencher. (**E**) Inhibition of RSV A2 replication in HeLa cell GFP reporter assay and APC-126 replicon assay by **22**. RSV eGFP EC_50_ = 2.28 µM, ATPLite CC_50_ > 50 µM, Replicon EC_50_ = 2.22 µM, Replicon CC_50_ > 50 µM. HeLa assays: 3,000 cells/well, rgRSV224 viral multiplicity of infection (MOI) = 1, 37°C, 5% CO_2_, 72 h. APC-126: 3,500 cells/well, 37°C, 5% CO_2_, 72 h. (**F**) Surface plasmon resonance (SPR) sensorgram for RSV-L+P binding to **22**. RSV L+P immobilized at 30 µg/mL, 1,200 s. **22** injected for 60 s, 30 µL min^−1^ flow rate, 500 s dissociation. *k*_a_ = 1.28 × 10^5^ M^−1^ s^−1^, *k*_d_ = 6.09 × 10^−2^ s^−1^, *K*_D_ = 0.47 µM. All data are representative of at least two independent experiments and plotted as mean ± SD.

Production of the large RdRp complex has proven difficult, as the yield of the full-length RSV L+P protein expression was low, and generating enough recombinant material to source an HTS was deemed extremely challenging (40). To overcome this, we employed a protein design strategy to generate a truncated construct by removing disordered segments. Cryo-EM structures of the L+P complex suggested that the N-terminal domain of the P protein was unstructured (Fig. 1A) (27, 31). Using this insight, a construct with an N-terminal truncation on the P protein, removing amino acids 1-124 (Δ1-124), was designed. The new full-length L and truncated P constructs improved the expression and stability of the L+P complex, enabling us to produce over 50 mg of protein for screening and hit triage.

Kinetic characterization of the truncated construct indicated that it was fit for purpose for screening. Reaction progress curves were linear over the course of 4 h, and initial velocities were linear with enzyme concentrations ranging from 3 to at least 20 nM (Fig. S2B and C). We determined the trailer complement RNA template (TrC-25) *K*_M_ value to be 0.11 ± 0.02 µM, which was comparable to the reported *K*_M_ value for the full-length enzyme (*K*_M_ = 0.23 ± 0.4 µM) using the same assay format (Fig. S2D). The apparent *K*_M_ value of pooled nucleotide substrates was determined to be 7.1 ± 1.1 µM with the truncated construct, which was fourfold lower than the full-length complex value of 28 ± 2 µM (Fig. S2E) (34). Observed rate of the reaction for the truncated construct was also reduced ~2.5-fold compared to the full-length protein (Fig. S2F), but reference compound IC_50_ values were similar against both complexes (Fig. S2G).

### High-throughput screening

To bias the HTS against less desired nucleotide-competitive inhibitors that would likely have poor antiviral activity due to high intracellular nucleotide concentrations (45), we opted to use 10 µM of each endogenous nucleotide (GTP, CTP, UTP), which is above their reported individual *K*_M_ values, and 100 µM ATPαS (34). The TrC-25 RNA template concentration was 5 µM, which was also above its *K*_M_ value determined in the luminescence assay. To minimize reagent consumption, the assay was miniaturized to a 1536-well format with a final reaction volume of 3 µL. At 20 nM RSV L+P, the assay signal-to-background was 2, and the robust Z’ value was consistently >0.6.

A small pilot screen of approximately 11,000 compounds had a high hit rate (>3.6%), which suggested that the HTS assay was highly susceptible to one or more false-positive mechanisms. To aid with triage, we tested if inhibition of the coupling system was the primary factor for the high hit rate by developing a counterscreen whereby PP_i_ was spiked into the reaction in the absence of RSV L+P. Approximately 80% of the hits were active in the counterscreen, suggesting that they were targeting either ATP sulfurylase or luciferase (Fig. S3A). Given that luciferase is frequently utilized for screening applications, we suspected that the sulfurylase was the vulnerable component. Filtering out false positives reduced the hit rate to 0.8% and provided a path forward for the full HTS campaign (Fig. S3B). In total, 24 K compounds showing >30% inhibition in the luminescence assay at 4.2 µM were tested in the counterscreen at the same concentration. Because a direct correlation between RSVpol potency and inhibition in the counterscreen could not be established, stringent selection criteria were implemented to remove false positives. Compounds showing <10% inhibition in the counterscreen or a % inhibition value that was fourfold higher in the luminescence assay were progressed to dose-response, and hits were tiered according to their potency and max percent effect in both assays (Fig. S3C).

Following resynthesis, the biochemical activity of compound **1** (Fig. 1B) was confirmed with a potency of 0.61 µM against the truncated construct and no activity in the counterscreen up to 100 µM (Fig. 1C). To confirm target engagement, a biophysical SPR assay was established using full-length RSV L+P complex containing an N-terminal His-tag on the P protein subunit. Here, the *K*_D_ value of **1** was determined to be 2.3 µM (sensorgram not shown), which was comparable to the IC_50_ value determined in the biochemical assay.

### Ligand-based approach and SAR

To validate the newly identified chemotype, we set out to explore its preliminary SAR. Keeping the spiro-oxindole core intact, analogs were designed to vary the southern vector R^1^, left-hand-side R^2^, and right-hand-side R^3^ (Fig. 6A).

To this end, the syntheses of compound **1** and its analogs commenced from Boc-protected spiro-oxindoles that were either commercially available (Fig. 6B; compounds **2**, **3**, **5**, **6**, **7**) or synthesized in one step (compounds **4** and **8**). Deprotection under acidic conditions afforded compounds **9** to **15**, which underwent nucleophilic aromatic substitution with 5-bromo-3-chloropicolinonitrile to yield target compound **1** and analogs **16** to **22**.

To incorporate additional variations of R^2^ and R^3^, 5-fluoro-oxindoles **19** (Fig. 6C) and **28** (Fig. 6D), respectively, were chosen as starting materials. Compared to the 5-bromo oxindoles, 5-fluoro analogs showed slightly increased solubility in organic solvents and allowed for easier handling and purification. Derivative **28** was also chosen to avoid undesired cross-reactivity in downstream Buchwald-Hartwig coupling. Thus, the common starting material **19** underwent nucleophilic substitution with the appropriate alkyl halide to afford analogs **23** to **25** bearing structurally differentiated left-hand groups. On the other hand, in order to generate a set of compounds with a different right-hand side, commercially available benzylated oxindole **26** was subjected to methylation and deprotection conditions to yield intermediate **28**. Nucleophilic aromatic substitution with the corresponding electron-deficient partner afforded **29** and **30**. Buchwald-Hartwig coupling (46, 47) of **28** with the appropriate aryl halides in the presence of RuPhos Pd G4 (48, 49) yielded compounds **31** to **33**, with the latter being subjected to further functionalization to give analog **34**.

Synthesized analogs were tested in the aforementioned luminescence assay to determine the inhibitory effect. To evaluate the potential translation of the biochemical activity into cellular systems, compounds were also tested in an eGFP-reporter phenotypic assay to measure inhibition of RSV infection of HeLa cells (36, 50). Compounds **16** to **19** showed that the 5-bromo of the oxindole could be replaced by halogens, lipophilic methyl, and polar methoxy groups, with the biochemical IC_50_ ranging from 1.03 to 3.10 µM (Table 1). In the phenotypic assay, however, poor cellular EC_50_ values ranging from 6.54 to 15.9 µM were observed, with compounds **16**, **18,** and **19** showing non-optimal cytotoxicity with E_max_ ranging between 45.3% and 82.4% (Table 1). A substituent at the 5-position of the oxindole was observed to be important for inhibition, since compound **20** showed loss of potency in the biochemical assay (IC_50_ = 6.63 µM, Table 1) and poor cellular activity (EC_50_ = 8.34 µM with CC_50_ > 3.80 µM at E_max_ = 42.9%, Table 1) compared to **1**.

**TABLE 1:** SAR of **1**

				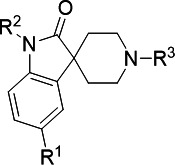						
**Cmpd**	**R^1^**	**R^2^**	**R^3^**	**Biochemical IC_50_ (µM)*^a^***	**Biochemical E_max_ (%**)	**Cellular EC_50_ (µM)*^b^***	**Cellular E_max_ (%**)	**Cytotoxicity CC_50_ (µM)*^c^***	**Cytotoxicity E_max_ (%**)	**HLM** **CL_int_*^d^***
1	Br	H	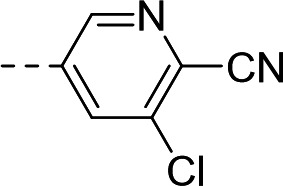	0.67	110	6.68	94.6	>3.80	63.1	<7.7
16	Cl	H	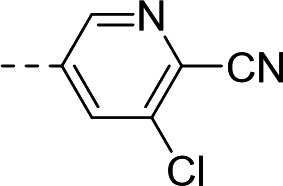	1.03	108	8.07	94.2	>13.0	45.3	<7.7
17	Me	H	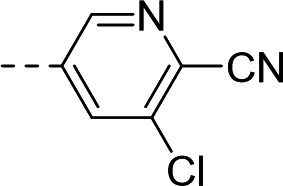	1.75	111	10.9	55.1	>50.0	8.6	123
18	OMe	H	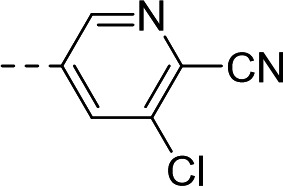	3.10	100	15.9	96.8	24.4	82.4	248
19	F	H	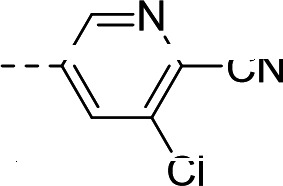	2.70	106	6.54	99.9	>50.0	52.2	19
20	H	H	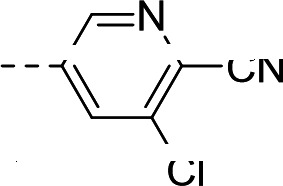	6.63	84.7	8.34	81.4	>3.80	42.9	19.7
23	F	Me	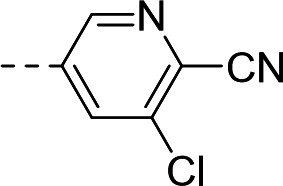	1.57	103	3.67	93.3	>3.74	55.7	19
24	F	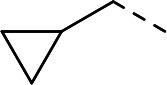	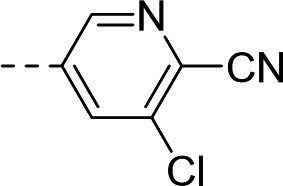	0.93	89.4	8.61	78.3	>7.77	51.8	46.3
25	F	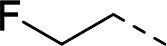	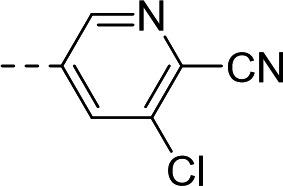	2.86	90.7	8.86	80.3	>23.4	66.5	14.1
21	F	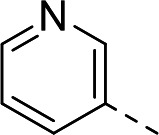	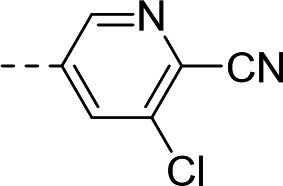	0.80	107	3.38	95.8	>2.71	44.8	213
29	F	Me	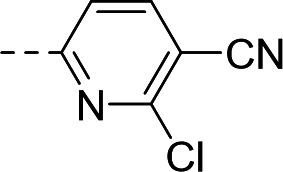	1.56	95.5	7.89	94.3	>8.43	66.9	31.9
30	F	Me	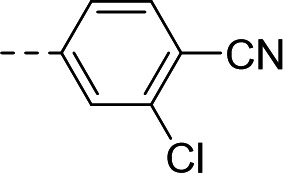	1.25	100	4.35	95.8	>6.65	58.0	39.1
31	F	Me	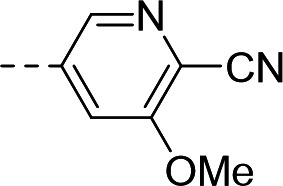	2.98	115	8.78	98.6	>22.7	37.6	20.5
32	F	Me	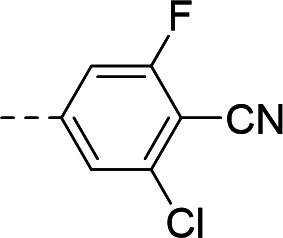	1.90	91.2	13.8	92.6	>5.78	50.9	64.8
34	F	Me	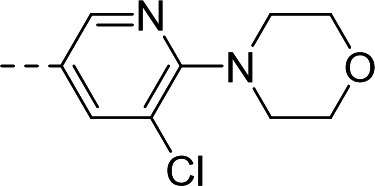	>30.2	69.5	30.3	49.6	>50.0	41.6	126
22	Br	Me	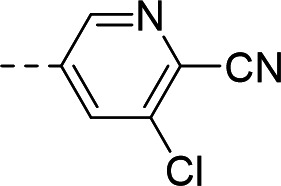	0.27	105	2.38	94.0	>50.0	12.8	10.9

^*a*^ Biochemical activity in pyrophosphate luminescence detection assay, IC_50_, 50% effective concentration (*n* ≥ 2).

^*b*^ Cellular activity in RSV eGFP phenotypic assay in HeLa cells, EC_50_, 50% effective concentration (*n* ≥ 2).

^*c*^ Cytotoxicity in HeLa cells, CC_50_, 50% cytotoxic concentration (*n* ≥ 2).

^*d*^ *In vitro* intrinsic clearance in human liver microsomes (HLM), values in µL/min/mg microsomal protein (*n* = 1).

Substitution of the left-hand side R^2^ was next investigated. Compounds **23** to **25** and **21** showed that both sp^3^ and sp^2^ characters could be tolerated in this vector, with the biochemical IC_50_ ranging from 0.80 to 2.86 µM (Table 1). The cellular EC_50_ ranged from 3.38 to 8.86 µM, but the E_max_ of CC_50_ curves (ranging between 44.8% and 66.5%) still indicated a poor specificity index (Table 1). When we turned our attention to explore R^3^, other pyridines were synthesized, such as 2-pyridine **29** or methoxy-substituted **31**. The electronics of R^3^ were also varied by introducing phenyls **30** and **32**. Overall, when the para-cyano group was retained, biochemical activities in the micromolar range were observed (IC_50_ values of **29** to **32** were between 1.25 and 2.98 µM, Table 1). The cellular activities remained moderate to poor, with EC_50_ between 4.35 and 13.8 µM and E_max_ of cytotoxicity between 37.6% and 66.9% (Table 1). In comparison, changing the para-cyano substituent to a morpholine in compound **34** was detrimental to inhibitory activity, highlighting the importance of the para-cyano group for potency. Across the board, the micromolar biochemical activities being retained across different vectors were encouraging as preliminary results for this chemotype. Future work would focus on improving the weaker activity in the cellular assay.

Initial readouts for intrinsic clearance in human liver microsomes were mostly in the acceptable range, with only four compounds **17**, **18**, **21**, and **34** showing low metabolic stability in human liver microsomes (Cl_int_ > 100 µL/min/mg microsomal protein, Table 1), highlighting another potential advantage for this chemotype. Taking into account both potency and metabolic stability parameters, we identified the best moiety from each vector for the first combination exercise, using R^3^ as 3-chloropicolinonitrile, R^2^ as methyl, and R^1^ as 5-bromo oxindole. Gratifyingly, the resulting compound **22** displayed improved biochemical potency (IC_50_ = 0.27 µM, Fig. 1D) which was confirmed by SPR (*K*_D_ = 0.47 µM, Fig. 1F). Moreover, contrary to the analogs discussed above, cellular activity in the antiviral assay (EC_50_ = 2.38 µM) was also accompanied by low cytotoxicity CC_50_ > 50.0 µM and a low E_max_ of 12.8%, equating to a specificity index >22 (Fig. 1E). In addition, compound **22** was similarly efficacious at inhibiting replication of clinically isolated RSV A and B strains as determined by quantitative reverse transcription PCR (RT-qPCR) (Table S1) (36). On-target activity was confirmed in the minireplicon assay (EC_50_ = 2.22 µM, Fig. 1E) and, at the same time, good intrinsic clearance in human liver microsomes was retained (Cl_int,h_ = 10.9 µL/min/mg microsomal protein, Table 1). This promising result suggested that this chemotype could be further optimized for cellular activities.

### Biochemical mechanism of action

**Fig 2:**
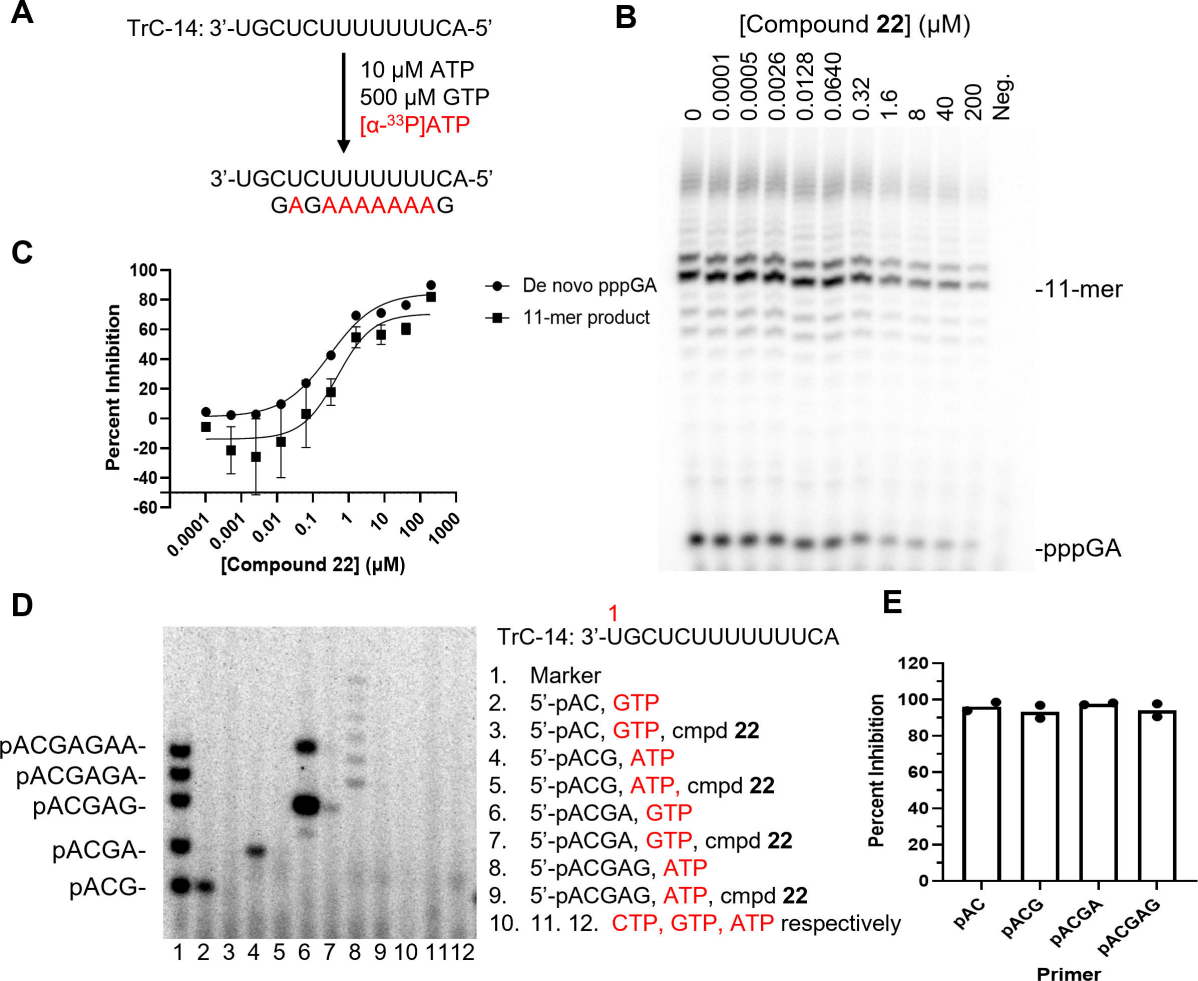
Inhibition of *de novo* initiation and primer extension of RSV L+P RNA synthesis by compound **22**. (**A**) Reaction scheme of *de novo* (starting from +3 site on TrC-14 template) initiation assay. (**B**) Representative sequencing gel image showing RNA products synthesized by RSV L+P at various concentrations of **22** (0.1 nM to 200 µM). The reactions contained 10 nM RSV L+P, 2 µM RNA template (TrC-14), 500 µM GTP, 10 µM ATP, and 170 nM [α-^33^P]ATP. RNA synthesis started from the +3 site and paused with a major 11-mer product. Negative control (Neg.) was the reaction without the enzyme. (**C**) Quantification and analysis of the inhibition of pppGA and the 11-mer RNA products. The data were fit to the Hill equation, and the inhibition of *de novo* pppGA formation exhibited an IC_50_ of 0.29 µM with a maximal inhibition of 85%, whereas inhibition of the 11-mer product exhibited an IC_50_ of 0.48 µM with a maximal inhibition of 71%. Data are representative of three technical replicates and plotted as mean ± SD. (**D**) Polyacrylamide sequencing gel showing single-nucleotide incorporation from a set of short primers with or without 50 µM **22** with TrC-14 template. (**E**) Inhibition of primer extension by **22** to primers with various lengths. Data were analyzed from the gel in D. Bars indicate the mean, and data points from two independent experiments are shown.

**Fig 3:**
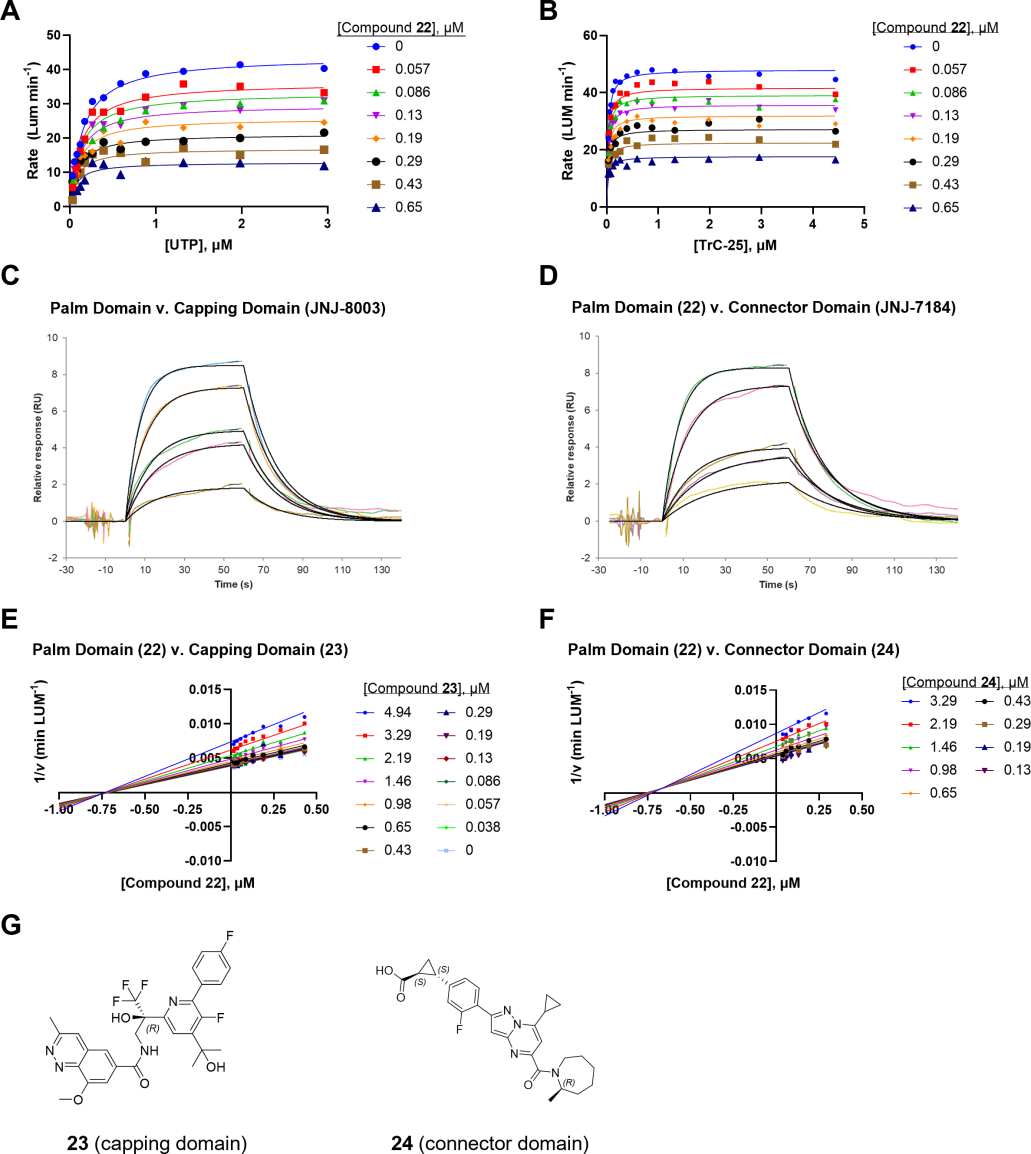
Compound **22** is an allosteric inhibitor of RSVpol. (A and B) The mode of inhibition for **22** was (**A**) mixed noncompetitive/uncompetitive (α = 0.33 ± 0.27) with respect to UTP and (**B**) noncompetitive (α = 1.1 ± 0.6) with respect to the TrC-25 RNA template. (C and D) SPR *K*_D_ determined for **22** in the presence of (**C**) capping domain binder JNJ-8003 (compound **22** *k*_a_ = 1.19 × 10^5^ M^−1^ s^−1^, *k*_d_ = 6.28 × 10^−2^ s^−1^, *K*_D_ = 0.53 µM) and (**D**) connector domain binder JNJ-7184 (compound **22** *k*_a_ = 1.40 × 10^5^ M^−1^ s^−1^, *k*_d_ = 7.25 × 10^−2^ s^−1^, *K*_D_ = 0.52 µM). (E and F) Yonetani-Theorell analysis showing that binding of **22** was not mutually exclusive with (**E**) compound **23** (γ = 1.1 ± 0.2) and (**F**) compound **24** (γ = 0.88 ± 0.72). (**G**) Chemical structures of capping **23** and connector **24** domain ligands.

To further investigate the pharmacology of compound **22**, a series of biochemical and biophysical assays were used to determine the mode of inhibition and mechanism of action. Compound **22** inhibited dinucleotide pppGpA formation from the +3 site on the TrC-14 RNA template with an IC_50_ value of 0.29 µM (Fig. 2A through C) and primer extension from AC, ACG, ACGA, and ACGAG (Fig. 2D and E) (36). It also inhibited primer extension from the +1 and +3 positions on the Le-11 RNA template (Fig. S4). This data suggests that compound **22** could inhibit RSVpol RNA synthesis at the *de novo* initiation and early elongation steps. To determine the mode of inhibition, progress curve analysis was carried out with the full-length complex. UTP was selected to represent nucleotide substrates since initial rates determined at varying UTP concentrations resulted in the best fit to the Michaelis-Menten equation. Compound **22** decreased V_max_ but had a negligible effect on the *K*_M_ of UTP (Fig. 3A). Fitting the data to a mixed model inhibition equation returned an α = 0.33 ± 0.27, suggesting that **22** was mixed noncompetitive/uncompetitive with respect to nucleotide substrates. Similarly, **22** decreased V_max_ with respect to TrC-25, but did not affect *K*_M_ (Fig. 3B). Statistical comparison of global fits indicated that a noncompetitive inhibition model was preferred with an α = 1.1 ± 0.6. Together, these data indicated that the binding site for compound **22** was distinct from the nucleotide and RNA binding sites.

### Binding site mutual exclusivity

To determine if compound **22** was acting at a known allosteric site, we measured binding affinity in the presence of competitor molecules, JNJ-8003 (capping domain inhibitor) (36) and JNJ-7184 (connector domain inhibitor) (24), using an SPR ABA method. Here, the competitor was held at a constant concentration (60 nM for JNJ-8003 and 150 nM for JNJ-7184) during baseline acquisition, association, and dissociation of the test compound. Dose-responsive binding of **22** was observed in the presence of the competitor molecules, indicating binding to a distinct site. Figure 3C and D represent the *K*_D_ evaluation by fitting five dose response points for the binding of **22** against RSV L+P-JNJ-8003 and RSV L+P-JNJ-7184 complex, respectively.

In parallel, we utilized Yonetani-Theorell mutual exclusivity analysis (51) to determine if our lead molecule can bind independently of the reported ligands, compound **23** (capping domain) (52) and compound **24** (connector domain) (Fig. 3G) (53). Here, matrix titrations of the two inhibitors were carried out, and initial rates were determined for each inhibitor combination. Data were fitted to the Yonetani-Theorell equation (Supp. equation S1) to determine the interaction constant, γ. When γ = 1, the two inhibitors can bind independently such that binding of one inhibitor does not affect binding of the other. When γ > 1, binding is antagonistic, and as γ approaches infinity, the ligands are considered mutually exclusive. Conversely, when γ < 1, binding is synergistic. When compound **22** was titrated in combination with the capping inhibitor **23**, a γ value of 1.1 ± 0.2 was obtained, indicating that the two inhibitors can bind independently of each other and that there was little energetic linkage between these two binding sites (Fig. 3E). Similarly, a γ value of 0.88 ± 0.72 was obtained with compound **22** and the connector domain inhibitor **24** (Fig. 3F).

### Compound 22 binds to a novel induced binding pocket

**Fig 4:**
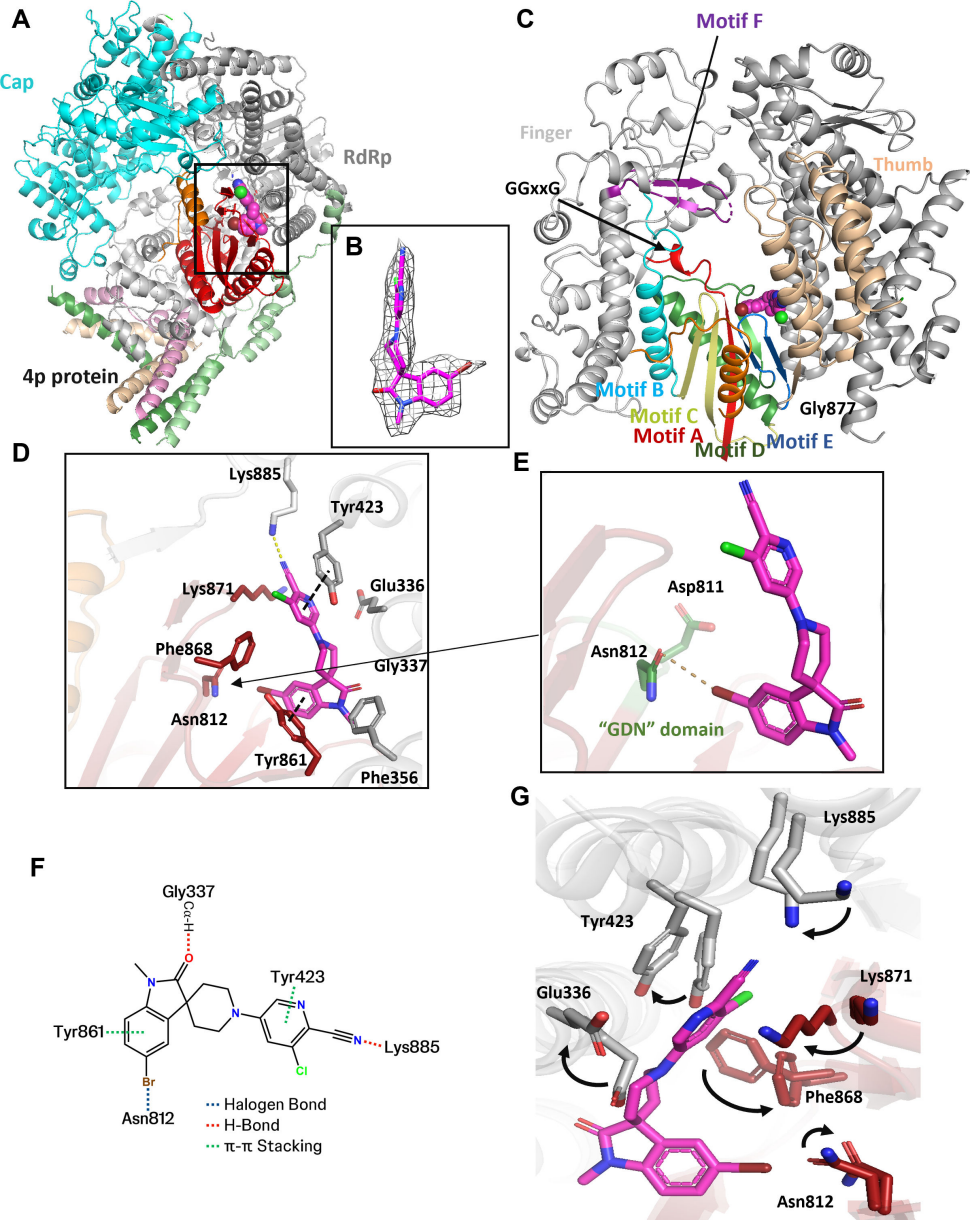
Structural basis of the interactions between RSVpol and compound **22** (PDB 9N36, EMD 48846). (**A**) Visualization of the Cryo-EM structure illustrating the RSVpol complex with compound **22**. The RdRp domain, capping domain, palm domain, four partial P proteins, and compound **22** are color-coded in gray, cyan, red, light green, pink, light orange, green, and magenta, respectively. (**B**) Depiction of the cryo-EM density corresponding to compound **22**. The ligand density is shown at a contour level of 0.339, as displayed in Chimera. (**C**) Presentation of the binding environment for compound **22**. (**D**) Binding pocket for compound **22**, with the compound and residues within 4 Å that form interactions shown in stick representation. (**E**) The interaction between compound **22** and the GDN motif. The GDN motif is shown as green sticks. (**F**) Two-dimensional interaction patterns of compound **22** and RSV L were highlighted using dashed lines. (**G**) Structural comparison of the ligand binding pocket between compound **22**-bound and Apo RSV L, featuring rotamer transitions for Lys885, Lys871, Phe868, Asn812, and Glu336, indicated by arrows.

To gain a comprehensive understanding of the interaction between **22** and RSVpol, we conducted a detailed structural analysis of RSV L+P complexed with compound **22** using cryo-EM. This led to a 3D reconstruction with a global resolution of 2.72 Å (Fig. 4A through C; Table S2 and Fig. S5). Subsequent analysis allowed us to model the RSV RdRp and capping domains, as well as four partial P proteins.

Within the map, we observed a distinct density corresponding to compound **22**, situated within a novel binding site adjacent to the palm subdomain of RdRp. This binding site (Fig. 4C through F) featured a deep hydrophobic pocket located in close proximity to motif E (comprising residues Met866-Asn876), which formed a β hairpin connecting the palm domain to the thumb domain of the polymerase. Compound **22** adopted an L-shaped configuration within this binding site, establishing significant interactions with nearby residues, as illustrated in Fig. 4D through G. Notably, the cyano group established a hydrogen bond with the ammonium group of Lys885. Initial SAR analysis underscored the importance of this interaction for potency, as substituting the cyano group resulted in inactive compound **34** (Table 1). The subsequent pyridine ring engaged in face-to-face π–π stacking with Tyr423. A pseudo-H-bond was observed between the indoline carbonyl oxygen atom and the Cα-hydrogen atom of Gly337. Hydrophobic interactions involved T-shaped Pi–Pi stacking of the indoline ring with Tyr861, as well as halogen bonding between the sidechain carbonyl oxygen atom of Asn812, a component of the conserved catalytic motif (Gly810-Asp811-Asn812 [GDN]), and the bromo atom. Halogen bonding contributed to binding, since replacing the bromo substituent with a proton in compound **20** gave a 10-fold loss in potency. In contrast, there were only a few contacts involving the central piperidine ring (Fig. 4F). Sequence conservation analysis within a 5 Å radius of **22** revealed that the pocket contact residues were completely (100%) conserved among RSV A and RSV B strain sequences (Fig. S6), consistent with the observed clade coverage (Table S1), and suggests the possibility of a pan-RSV targeting approach.

Comparative analysis of the ligand binding site in the absence (Apo, PDB 6PZK) and presence of **22** revealed significant conformational changes, particularly involving the side chains of Glu336, Met340, and Tyr423 (Fig. 4G). In the Apo structure, these side chains exhibited steric clashes when superimposed with the compound **22**-bound conformation, necessitating a reorganization to accommodate the observed binding mode. Additionally, the side chain of Phe868 moved closer to facilitate a hydrophobic interaction with compound **22**. Major conformational rearrangements were observed in the side chains of Lys885, Lys871, and Asn812 upon ligand binding, enabling interactions with **22**. The conformational changes that occurred upon ligand binding highlight the dynamic nature of this novel induced fit binding pocket.

### Stable β-sheet and helix formation induced by the binding of compound 22

The palm domain of RSVpol comprises beta hairpins formed by motifs A, B, C, D, and E (54). The binding pocket for compound **22** was composed of the beta sheet from motif A, motif E, and motif D within the Palm domain, and anti-parallel α-helices of RSV RdRp. Specifically, motif E, which includes two beta sheets and a loop, is directly involved in the formation of the ligand-binding pocket.

**Fig 5:**
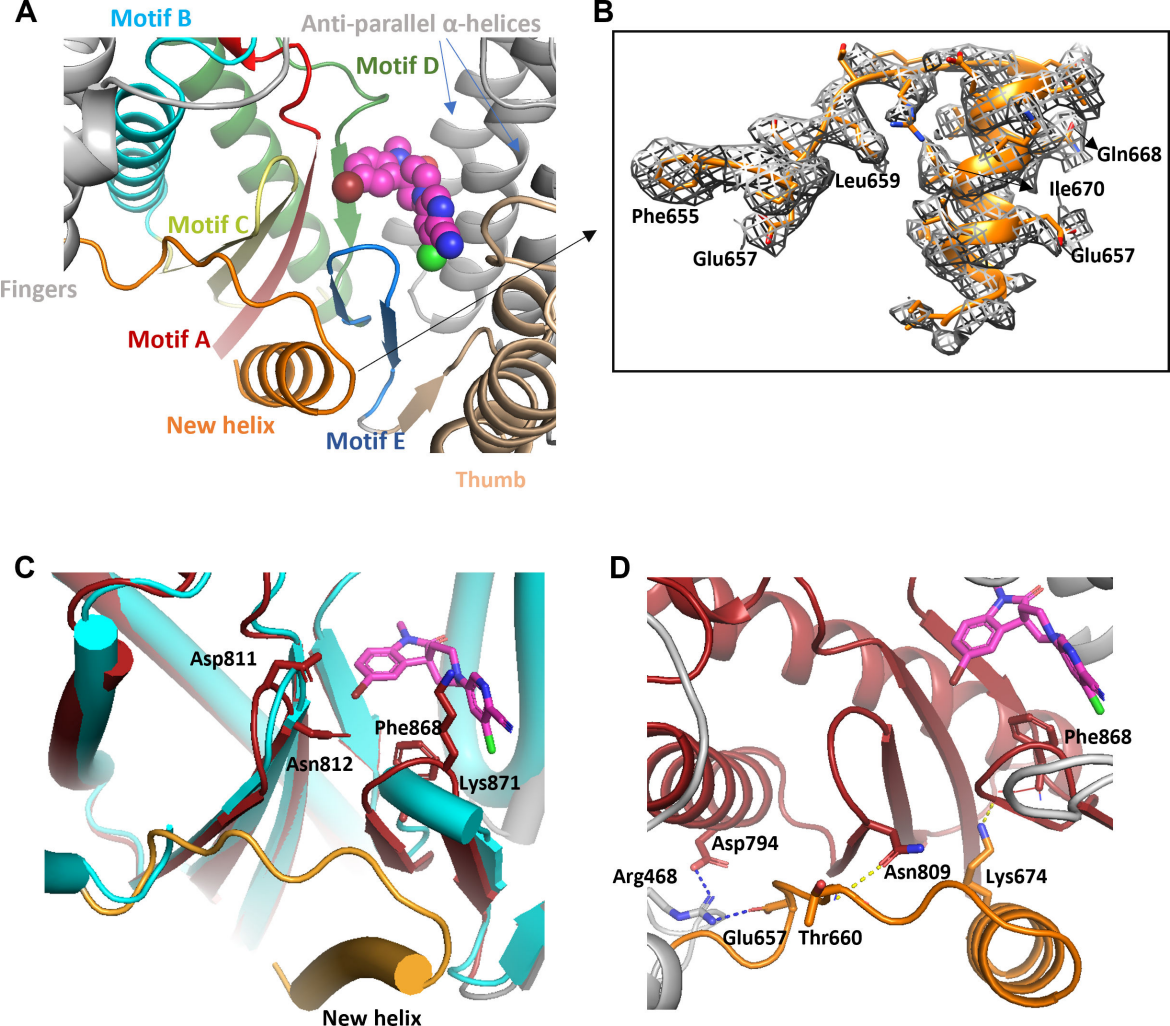
Detailed insights into the structural features and interactions within the compound **22** binding domain of RSVpol. (**A**) Compound **22** within the RSVpol domain is surrounded by alpha-helical and beta-sheet bundles. The alpha helix and beta strand, significantly stabilized by compound **22**, are highlighted in orange and blue, respectively. (**B**) Raw density visualization of the newly stabilized alpha helix. The new helix density is shown at a contour level of 0.245, as displayed in Chimera. (**C**) Comparison of compound **22** bound and the Apo form (PDB: 6PZK). In the compound **22** bound form, the palm domain and new helix are depicted in red and orange, respectively, while the Apo form is displayed in cyan. Compound **22** is shown as magenta. (**D**) Depiction of key interactions surrounding the helix that contribute to its stabilization. The color code in panels C and D in compound **22** bound form corresponds to that in Fig. 4A.

Upon thorough examination of all density maps, we have identified additional unresolved density that was not present in the Apo cryo-EM structure (Fig. 5A through D). This density corresponded to a helix and loop region spanning residues 655 to 677, suggesting significant flexibility in the absence of ligand binding. However, this flexibility was notably stabilized upon ligand binding. This finding is corroborated by protection induced by compound **22** binding in this region in corresponding hydrogen-deuterium exchange coupled with mass spectrometry (HDX-MS) experiments (Fig. S7). The new helix itself did not directly interact with compound **22**; its stabilization relied on specific beta sheets of motifs A, C, and E, which likely formed the cavern, and the new helix was inserted into the cave. Moreover, the newly identified helix was primarily stabilized through the formation of hydrogen bonds and salt bridges with adjacent key residues. Specifically, Arg468 formed salt bridges with Asp794 and Glu657, while a hydrogen bond was established between Asn809 and the Thr660 main chain of the new helix (Fig. 5D).

## DISCUSSION

While Apo and RNA-bound RNA polymerases of various non-segmented negative-strand RNA viruses share a broadly similar architectural framework, the structural characteristics of non-nucleotide compound-bound RSV polymerases have remained elusive until recently (36, 40, 41). Our research unveiled and characterized a novel NNI for RSVpol and presented the first cryo-EM structures of this compound bound to a previously undescribed site in the palm domain.

Truncation of the disordered region of the P protein improved expression yields and enabled HTS that identified compound **1** as a submicromolar inhibitor of RdRp polymerase with no activity against the human mitochondrial RNA polymerase. To validate this chemotype, preliminary SAR campaigns focusing on three vectors were carried out. Combination of the most promising substituents afforded compound **22**, which not only showed improved biochemical activity and biophysical binding, but also exhibited antiviral activity with good SI and metabolic clearance. Interactions observed between **22** and the palm pocket pave the way for additional structure-based drug design. Given the importance of the cyano-lysine interaction for inhibition, strengthening this interaction by shortening the distance could be advantageous. Furthermore, compared to the Apo structure, WAT1 in the Holo structure (Fig. S8) displayed increased excess free energy of binding with both enthalpic and entropic components, suggesting that the placement of lipophilic or polar substituents on the pyridine to displace this high-energy water molecule could improve binding affinity.

Our investigation demonstrated that the binding of compound **22** to RSVpol induced specific conformational changes, resulting in the formation of an induced binding pocket. Unlike the binding pocket of JNJ-8003, **S1**, and MRK-1 on the capping domain (Fig. S1), this induced pocket relied on specific anti-parallel β-sheets from motifs A, C, D, and E (Fig. 4; Fig. S9). A similar inhibitor binding pocket was also observed with the HCV polymerase (55–57). Structural comparison between HCV and RSV polymerases revealed an RMSD of approximately 18.326 Å (Fig. S9). Despite differences in protein sequence, the inhibitor binding pockets in RSV and HCV polymerase show significant structural similarity. In HCV, ligand binding involves a deep hydrophobic pocket adjacent to motif E, forming a β-hairpin known as the primer grip site (56, 57). Drawing parallels, binding to this site could inhibit RSV initiation and elongation, which was confirmed biochemically (Fig. 2; Fig. S4). In biochemical assays focusing on *de novo* initiation, compound **22** displayed mixed noncompetitive/uncompetitive behavior with respect to UTP and noncompetitive behavior with respect to the RNA template (Fig. 3). This is in contrast to the HCV inhibitor, which displayed mixed noncompetitive/competitive inhibition with respect to GTP, suggesting the potential for class switching at this allosteric site (55). Taken together, these data suggest that compound **22** can stabilize an inactive conformation of the polymerase, possibly by changing the orientation of Asn812 in the catalytic GDN motif (Fig. 4G). This is consistent with the observed mode of inhibition and with the ability of compound **22** to interfere with both *de novo* initiation and elongation.

When superimposing the compound **22**-bound RSVpol onto Apo RSVpol, a notable difference was observed in the RdRp domain of the RSV L protein: the supporting helix and supporting loop, which are missing in the Apo structure due to their flexibility, became clearly defined. Another analysis comparing Le10-bound RSVpol (58) with compound **22**-bound RSVpol revealed a striking similarity in their overall structural configuration (Fig. S10). Despite differences in the position of the ligand binding site and RNA synthesis catalytic pocket, the supporting helix and supporting loop were well stabilized by either ligand or RNA binding. Compared to the RNA-bound structure (58), two key residues contributing to the RNA template stability, Lys783 of the palm and Thr660 from the supporting loop, exhibited side chain movement in our structure to accommodate ligand binding. The functional relevance of supporting helix and supporting loop was underscored by a report from another group (58) that indicated the supporting loop stabilized the initial nucleotide of the Le promoter. Contrasting observations arise from the structure of the EBOV polymerase complex (59), where the first three nucleotides of RNA reside within the catalytic pocket without interacting with the supporting loop or helix. Further comprehensive studies are required to fully elucidate the precise functions of these two novel structural elements. The chemotype identified herein provides a useful tool for investigating the structure and function of the newly identified palm domain binding site and could serve as a lead for the development of a novel class of allosteric NNI therapeutics against RSV.

## MATERIALS AND METHODS

### Cloning and expression of the RSV L+P complex

Codon-optimized human RSV L protein with an N-terminal Twin-Strep-tag and either full-length RSV P (1-241) protein with a C-terminal 6xHis-tag or untagged, truncated RSV P (125-241) protein were subcloned into the pFastBac Dual vector (Gibco), and baculovirus-infected insect cells (BIIC) were prepared using standard procedures. The L+P complex was expressed in Sf9 cells by infecting with the recombinant BIICs at a multiplicity of infection (MOI) of 0.5 and 5 pfu/cell for full-length and truncated L+P complexes, respectively, and harvested 72 h post-infection.

### Purification of the RSV L+P complex for screening

Cells were lysed using Dounce homogenization followed by one pass through a microfluidizer in Lysis Buffer (50 mM Tris-HCl pH 8.0, 300 mM NaCl, 10% glycerol, 1 mM TCEP) supplemented with 1 mM AEBSF (MP Biomedicals), cOmplete EDTA-free Protease Inhibitor Cocktail (Roche), and TurboNuclease (Accelagen). Cellular debris was cleared using high-speed centrifugation, and the soluble fraction was loaded onto a StrepTrap XT column (Cytiva) equilibrated in Strep Buffer A (50 mM Tris-HCl pH 8.0, 300 mM NaCl, 10% glycerol, 1 mM TCEP, 0.05% CHAPS) supplemented with cOmplete EDTA-free Protease Inhibitor Cocktail. Bound protein was eluted using 50 mM Biotin (Sigma-Aldrich) in Strep Buffer A. Fractions containing L+P complex were combined, diluted with a half volume of Heparin Buffer A (50 mM Tris-HCl pH 8.0, 10% glycerol, 1 mM TCEP, 0.01% CHAPS), and further purified on a HiTrap Heparin HP column (Cytiva) equilibrated in Heparin Buffer A containing 200 mM NaCl. Bound protein was eluted using a gradient from 200 to 500 mM NaCl over 15 column volumes. Fractions containing the eluted L+P complex were combined and concentrated to 0.5–1 mg/mL using an Amicon Ultra concentrator with a 100 kDa cutoff. The concentrated sample was flash frozen and stored at −80°C. The quality of the purified proteins was analyzed using SDS-PAGE under reducing conditions.

### Purification of the RSV L+P complex for cryo-EM

Cells expressing the full-length RSV L+P complex were lysed using Dounce homogenization followed by one pass through a microfluidizer in Lysis Buffer (50 mM Tris-HCl pH 8.0, 300 mM NaCl, 10% glycerol, 1 mM TCEP) supplemented with 1 mM AEBSF (MP Biomedicals), cOmplete EDTA-free Protease Inhibitor Cocktail (Roche), and 50 units/mL TurboNuclease (Accelagen). Cellular debris was cleared using high-speed centrifugation, and the soluble fraction was loaded onto a StrepTrap XT column (Cytiva) equilibrated in Strep Buffer A (50 mM Tris-HCl pH 8.0, 300 mM NaCl, 10% glycerol, 1 mM TCEP) supplemented with cOmplete EDTA-free Protease Inhibitor Cocktail. Bound protein was eluted using 50 mM Biotin (Sigma-Aldrich) in Strep Buffer A. Fractions containing L+P complex were combined, diluted with a half volume of Heparin Buffer A (50 mM Tris-HCl pH 8.0, 10% glycerol, 1 mM TCEP), and further purified on a HiTrap Heparin HP column (Cytiva) equilibrated in Heparin Buffer A containing 200 mM NaCl. Bound protein was eluted using a gradient from 200 to 500 mM NaCl over 15 column volumes. Fractions containing the eluted L+P complex were combined, supplemented with NaCl to bring the final NaCl concentration to 500 mM, and concentrated using an Amicon Ultra concentrator with a 100 kDa cutoff. The concentrated sample was further purified on a HiLoad 16/600 Superose 6 pg column (Cytiva) equilibrated in 50 mM Tris-HCl pH 8.0, 500 mM NaCl, 5% glycerol, 1 mM TCEP buffer. Peak fractions were combined and concentrated to 1 mg/mL using an Amicon Ultra concentrator with a 100 kDa cutoff. The concentrated sample was flash frozen and stored at −80°C. The quality of the purified proteins was analyzed using SDS-PAGE under reducing conditions.

### Cloning, expression, and purification of POLRMT

Amino acids corresponding to residues 103-1230 of human POLRMT (hPOLRMT), preceded by a Tobacco Etch Virus protease-cleavable 10-Histidine tag, were cloned into the pET28a vector for overexpression in BL21 (DE3) *E. coli* cells. Overnight cultures of BL21 cells, cultivated in LB media, were used to initiate larger cultures (10 mL in 1 L). When the cultures reached an OD600 of 0.5, the temperature was lowered to 16°C, and protein overexpression was induced with the addition of 0.5 mM IPTG. The cells were harvested by centrifugation (3,000 × *g* for 30 min) and resuspended in a lysis buffer composed of 50 mM HEPES pH 8.0, 500 mM NaCl, 10% glycerol, 0.5 mM TCEP, 20 mM imidazole, 10 mM MgCl_2_, and 10 mM ATP. After performing three rounds of microfluidization at 15,000 psi while on ice, the cell lysate was centrifuged (33,000 × *g* for 30 min) to eliminate intact cells and cellular debris. hPOLRMT was subsequently purified from the supernatant through three sequential chromatographic methods: IMAC (Ni-NTA Excel, Cytiva), Heparin (Heparin HP, Cytiva), and gel-filtration chromatography (Superdex 200 Increase, Cytiva). The final storage buffer, which was also used for gel filtration, contained 50 mM HEPES pH 8.0, 500 mM NaCl, 10% glycerol, 0.5 mM TCEP, and 0.5 mM EDTA.

### Pyrophosphate luminescence detection assay

Reactions were run at a final volume of 3 µL for 90 min (full-length) or 3.5 h (truncated P protein) in complete assay buffer consisting of 50 mM Tris pH 7.5, 8 mM MgCl_2_, 10% glycerol, 5 mM DTT, 125 µg/mL BSA, and 0.1 U/µL RNaseIn (Promega) (34). Working solutions (3×) of RSV L+P (60 nM), substrate (30 µM each of GTP, CTP, UTP, 300 µM ATPαS [Jena Bioscience], 15 µM RNA template TrC-25), detection reagents (6 nM Luciferase, 900 µM luciferin, 3 U/µL ATP Sulfurylase [BioVision], 15 µM adenosine phosphosulfate [APS], 3 mM coenzyme A), were prepared in complete assay buffer. Compounds were dissolved in dimethyl sulfoxide (DMSO) and dispensed to white, 1536-well cyclic olefin copolymer (COC) plates (Corning, 9145BC) using an Echo Acoustic Liquid Handler (Beckman Coulter, San Jose, CA). Substrate (1 µL) and detection reagents (1 µL) were dispensed using a Multidrop Combi Dispenser (ThermoFisher, Waltham, MA), followed by enzyme (1 µL) to initiate the reaction. Final screening concentrations were 20 nM RSV L+P, 10 µM GTP/CTP/UTP, 100 µM ATPαS, 5 µM TrC-25, 2 nM Luciferase (Promega), 300 µM luciferin (Promega), 1 U/µL ATP sulfurylase, 5 µM APS, and 1 mM CoA. Plates were sealed with TopSeal (PerkinElmer, 6050185), centrifuged at 1,000 rpm for 1 min, and incubated away from light. Luminescence was detected using the PHERAStar FSX (BMG Labtech, Ortenberg, Germany). Data were normalized to vehicle and no enzyme controls and fit to a four-parameter non-linear regression equation using Genedata Screener to calculate an IC_50_. Figures were prepared with GraphPad Prism (GraphPad Software, version 9 for Windows, San Diego, CA). TrC-25 (5′-UAGUUUUUGACACUUUUUUUCUCGU-3′) was custom ordered from Dharmacon.

### Pyrophosphate detection counterscreen

To determine if compounds were interfering with the sulfurylase or luciferase in the pyrophosphate luminescence detection assay, a counterscreen was developed to monitor activity of the coupling system. Working solutions (3×) of substrate and detection reagents were prepared as before and added to 1536-well white, COC plates containing compound titrations. Then, 1 µL of a 3× solution of 4.5 µM sodium pyrophosphate (Sigma, P8010) was dispensed with the Multidrop Combi to initiate the reaction. Plates were sealed, centrifuged at 1,000 rpm for 1 min, and incubated away from light for 30 min. Luminescence was detected using the PHERAStar FSX. Data were normalized to vehicle and no pyrophosphate controls and fit to a four-parameter non-linear regression equation using Genedata Screener to calculate an IC_50_. Figures were prepared with GraphPad Prism.

### POLRMT assay

Working solutions (2×) of enzyme (16 nM) and substrate (200 nM FAM-HMRP template, 200 nM 6AG-BHQ, 200 nM HMRP primer, 9 µM ATP, 1.7 µM GTP, 0.20 µM CTP, 1.2 µM UTP) were prepared in complete assay buffer consisting of 50 mM HEPES pH 7.5, 2 mM MgCl_2_, 1 mM EGTA, 1 mM DTT, 0.1 U/µL RNaseIn, 200 µg/mL BSA, and 0.01% Pluronic F-127. Compounds were acoustically dispensed in DMSO to black, 1536-well non-treated COC plates (Corning, 9146BC), and then 2 µL of the 2× enzyme solution was added with a Multidrop Combi. Following a 30 min incubation at RT, 2 µL of the 2× substrate solution was added with a Multidrop Combi. Plates were centrifuged at 1,000 rpm for 1 min, covered, and incubated away from light for 1 h at RT. Fluorescence was detected using the PHERAStar FSX (Ex/Em 485/520 nm). Data were normalized to vehicle and no enzyme controls and fit to a four-parameter non-linear regression equation in Genedata Screener to calculate an IC_50_. Figures were prepared with GraphPad Prism. FAM-HMRP ssDNA template (5′-6-FAMCTCTCTCTCTCTATTACGTTGGCGCG-3′) and 6AG-BHQ ssDNA quencher (5′-AGAGAGAGAGAGBHQ_1-3’) were custom ordered through IDT. The HMRP RNA primer (5′-UUUUGCCGCGCCA-3′) was custom ordered from Dharmacon.

### SPR assay

A Biacore Series S SA (Cytiva) chip is first derivatized by a 60 s injection of 50 µg/mL Tris NTA-biotin (synthesized in-house). The chip was further conditioned by a 60 s injection of 350 mM EDTA (Cytiva, 28995043) followed by a 60 s injection of 0.5 mM NiCl_2_ (Cytiva, 28995043). Purified human, recombinant His-tagged RSV L+P protein (C-terminal 8× His) was captured via a 1,200 s injection at 30 µg/mL to obtain immobilization levels of 8,000–9,000 RU. The surface was then blocked with a 60 s injection of 100 µM PEG-biotin (Thermo Scientific). The experiments were performed at 25°C, using a Biacore 8K+ (Cytiva) and running buffer: 10 mM HEPES, 300 mM NaCl, 10 mM MgCl_2_, pH 7.4, 5% glycerol, 2% DMSO, 0.005% p20, 0.5 mM TCEP. Compound **22** was injected for 60 s at 30 µL/min, followed by a dissociation phase of 500 s using the parallel kinetics mode with an 8-point twofold dilution series with a top concentration of 10 µM. Reference and buffer-blank subtracted data were analyzed with the Biacore 8K Insight Evaluation Software and fit to a 1:1 Langmuir model to obtain *K*_D_ values and binding kinetics.

The Biacore A-B-A injection method was used to assess compound binding competition. RSVpol was immobilized as above. The protein surface was first saturated with competitor compound in the A injection, followed by varying concentrations of compound **22** in the 60 s B injection. Dissociation of any bound **22** was then measured for 500 s in the presence of a competitor. The temperature was 25°C, and the flow rate was kept at 30 µL/min. Compound **22** was injected for 60 s at 30 µL/min, followed by a dissociation phase of 500 s using a parallel-cycle kinetics mode with an 8-point twofold dilution series with a top concentration of 5 µM.

### *De novo* RNA synthesis assay

A reaction mixture consisting of 10 nM RSV L+P, 2 µM RNA template (TrC-14), 500 µM GTP, 10 µM ATP, and 170 nM [α-^33^P]ATP (3,000 Ci/mmol) tracer was incubated with varying concentrations of compound **22** or vehicle (5% DMSO) in buffer (50 mM Tris pH7.4, 8 mM MgCl_2_, 6 mM DTT, and 10% glycerol) for 1 h at 30°C (36). The reaction was quenched by an equal volume of gel loading buffer consisting of 90% formamide and 50 mM EDTA. Samples were heated to 95°C for 5 min, resolved on a 22.5% polyacrylamide urea sequencing gel at 80 W, and then transferred to Whatman cellulose chromatography paper (Millipore Sigma). The gel was dried for 1 h on a gel dryer (Bio-Rad), exposed to a phosphor screen, and imaged using a Typhoon phosphorimager (GE Healthcare). Data were quantified with ImageQuant (GE Healthcare), normalized to untreated and no enzyme controls, and fit to a four-parameter non-linear regression equation where IC_50_ represents the concentration of compound leading to a 50% decrease in product production.

### Single-nucleotide incorporation assays

A mixture of 0.2 µM RSV L+P, 0.2 µM RNA template (TrC-14), and 400 µM primer (sequences indicated in figure) was incubated in buffer consisting of 50 mM Tris pH 7.4, 2 mM DTT, and 6 mM MgCl_2_ with either 50 µM compound **22** or vehicle (5% DMSO) at 30°C for 10 min (36). Reactions were initiated by the addition of 100 nM [α-^32^P]NTP (3,000 Ci/mmol) tracer and run for an additional 30 min before quenching with an equal volume of gel loading buffer (90% formamide, 50 mM EDTA, 0.05% Xylene Cyanol and Bromophenol Blue). Gel images were obtained and processed as described for the *de novo* assay, and data were normalized to untreated controls to determine percent inhibition.

### HeLa cell rgRSV224 viral proliferation and cytotoxicity assays

Compound serial dilutions (9 point, fourfold) were prepared in DMSO, and 200 nL was transferred to black, 384-well, clear-bottom microtiter plates (Corning, Amsterdam, The Netherlands) via acoustic drop ejection (36, 50). To initiate the assay, 20 µL of culture media (RPMI medium without phenol red, 10% FBS-heat inactivated, 0.04% gentamycin) containing rgRSV224 virus with a GFP reporter (in-licensed from NIH, Bethesda, MD) (60) at an MOI of 1 was added with a Multidrop Combi. Then, 20 µL of a HeLa cell suspension (3,000 cells/well) was added with a Multidrop Combi, and cells were incubated at 37°C in a 5% CO_2_ atmosphere. After 72 h, GFP expression was quantified using an EnVision Multimode Plate Reader (Perkin Elmer, Zaventem, Belgium), and data were normalized to untreated and media-only (no virus) controls. Cytotoxicity was determined in parallel using the ATPlite kit (Perkin Elmer, Zaventem, Belgium). Luminescence was measured in white, 384-well microtiter plates (Corning) on a ViewLux uHTS Microplate Imager apparatus (PerkinElmer, Zaventem, Belgium), and cellular ATP content was quantified by normalizing to untreated and media-only (no cell) controls. Data were fit to a four-parameter non-linear regression equation in Genedata Screener, where EC_50_ and CC_50_ were defined as the concentration of inhibitor resulting in 50% efficacy and cytotoxicity, respectively. Figures were prepared with GraphPad Prism.

### APC-126 minireplicon and cytotoxicity assays

A proprietary BHK-based cell line, APC-126, harboring the RSV replicon, was obtained from Apath (New York, USA) and cultured in DMEM/Ham’s F-12 50/50, supplemented with 10% (vol/vol) FBS (Gibco), 1% (vol/vol) penicillin-streptomycin (Gibco), 1% (vol/vol) MEM non-essential amino acids (Gibco), 5% (vol/vol) Tryptose Phosphate Broth (Sigma-Aldrich), and 10 µg/mL blasticidin (Invitrogen) at 37°C in a humidified 5% CO_2_ incubator (36). Cells were plated (3,500 cells/well) in white, 96-well, clear-bottom plates (Corning) and incubated for 24 h. Compound serial dilutions (9 point, threefold) were prepared in DMSO and diluted 10-fold in culture media before treating cells (final [DMSO] = 1% [vol/vol]). After a 72 h incubation, luminescence was quantified using the Renilla-Glo Luciferase Assay System (Promega) on a VictorX3 plate reader (Perkin Elmer), and data were normalized to untreated and media-only (no cells) controls. In parallel, cytotoxicity was measured using CellTiter-Glo (Promega) under the same conditions. Normalized data were fit to a four-parameter non-linear regression equation in Genedata Screener, where EC_50_ and CC_50_ were defined as the concentration of inhibitor resulting in 50% efficacy and cytotoxicity, respectively. Figures were prepared with GraphPad Prism.

### Inhibition of RSV-A and B strains

Compound serial dilutions (9 point, fourfold) were prepared in culture media (RPMI-1640 supplemented with 10% fetal calf serum, 25 mM HEPES, 10 mM L-glutamine, and 0.02 µg/mL gentamycin), and 50 µL was transferred to black, 96-well microtiter plates (36). Then, 100 µL of a HeLa cell suspension was added to each well (5,000 cells/well), followed by 50 µL of the respective virus dilution as previously described (36). Cells were incubated for 72 h at 37°C in a 5% CO_2_ atmosphere. Then, the supernatant was removed, cells were washed twice with 100 µL cold PBS, and plates were sealed and stored at −80°C. Cells were lysed using the Ambion Cell-to-C_T_ kit, and lysates were placed on ice prior to RT-qPCR. Lysate (5 µL) was added to 0.27 µM reverse primers (RSV-A, RSB-B, or β-actin) and denatured at 75°C for 5 s. cDNA synthesis was carried out in Expand High Fidelity PCR Buffer with 3.5 mM MgCl_2_, 1 mM dNTP, 1 U/µL RNaseIn, and 1 U/µL Expand Reverse Transcriptase (Roche) by incubating at 42°C for 30 min, followed by denaturation at 95°C for 5 min. qPCR was carried out in 1× master mix (Roche) with 0.3 µM each of the forward and reverse primers, 0.1 µM probe (RSV-A, RSV-B, or β-actin), and 5 µL cDNA product on a Roche LightCycler480 (denaturation: 95°C for 600 s, 45 cycles: 95°C for 15 s (denaturation) and 60°C for 60 s (annealing/elongation), cooling: 40°C for 10 s). EC_50_ values were calculated from the corresponding Cp values by graphic interpolation and defined as the concentration of inhibitor required for a twofold reduction in RNA content relative to the untreated control. Primer and probe sequences were previously reported (36).

### Sample preparation for electron microscopy

The QuantiFoil Au 1.2/1.3 300 mesh grids underwent glow discharge using the PELCO easiGlow Discharge Cleaning System. The protein complex (3 µL, 0.8 mg/mL) was applied to the electron microscopy (EM) grids, followed by vitrification using the Vitrobot (Thermo Fisher Mark IV) with the following settings: blot time 5 s, blot force 0, wait time 0 s, inner chamber temperature 4°C, and 100% relative humidity. Flash-freezing in liquid ethane cooled by liquid nitrogen was performed. For the compound **22**-bound RSVpol complex, Cryo-EM data collection was automated on a 200 kV Thermo Scientific Glacios microscope controlled by the EPU software. Micrographs were acquired at 105,000× magnification using a Falcon4 detector (Gatan) in counting mode. Each 6 s exposure recorded 40 frames with a total dose of 40 e-/Å^2^. The calibrated physical pixel size for all digital micrographs was 0.948 Å. A total of 14,890 images were acquired. All details are summarized in Table S2.

### Cryo-EM image processing and three-dimensional structure reconstruction

Cryo-EM data collection and image quality assessment were conducted using cryoSPARC Live v3.2 installed on a local workstation. Concurrently, image preprocessing procedures, including patch motion correction, patch contrast transfer function estimation, blob particle picking (100–200 Å diameter), and extraction, were executed. Suitable two-dimensional (2D) classes were employed as templates for particle repicking. Following one round of live 2D image classification, approximately 2.7 million high-quality particle images were obtained. These selected particles were subsequently utilized for 3D reconstruction.

The initial iteration involved the generation of four starting 3D models, ultimately yielding a predominant 3D class. The primary class underwent non-uniform 3D refinement and local refinement, resulting in the refinement of 1,546,578 particles and the production of a 3D map with an average resolution of 2.72 Å (Fig. S5). Comprehensive statistical information regarding the cryo-EM experiments is presented in Table S2.

### Cryo-EM model building, refinement, and validation

The apo RSV L+P complex (PDB: 6PZK) served as the initial template for model generation. The model was meticulously docked into the EM density map using Chimera (61), followed by manual adjustments in Coot (62). A new helix was constructed manually in Coot, guided by the local density information. Validation of the geometry parameters for the final models was carried out in Coot, MolProbity (63), and EMRinger (64). The refinement process iteratively continued until no further enhancements were observed. Detailed statistics resulting from the final refinement are presented in Table S2.

To assess model overfitting, refinement against one cryo-EM half map was conducted. Fourier Shell Correlation curves were computed between the resultant model and the working half map, as well as between the resultant model and the free half and full maps for cross-validation. Figures were generated using PyMOL (The PyMOL Molecular Graphics System, Version 2.0 Schrödinger, LLC.) and Chimera.

### HDX-MS analysis

HDX-MS analysis used in this study followed the experimental workflow outlined elsewhere (36). Briefly, sequence coverage for RSVpol was generated using duplicate undeuterated controls. A 10 µL of 1.2 µM sample in Buffer B (20 mM HEPES, pH 7.5, 500 mM NaCl, 5% glycerol, 1 mM TCEP) was diluted with 60 µL of ice-cold quench (100 mM glycine, 7.04 M guanidine-HCl, 20 mM TCEP, pH 2.4) and followed by further dilution in 180 µL dilution buffer (100 mM glycine, 20 mM TCEP, pH 2.4). Samples were then injected into a Waters HDX nanoAcquity UPLC (Waters, Milford, MA) fitted with an in-line protease XIII/pepsin column (NovaBioAssays). Digested fragments were trapped on an Acquity UPLC BEH C18 peptide trap and separated on an Acquity UPLC BEH C18 column. A 10 min, 5% to 35% acetonitrile (0.1% formic acid) gradient at 60 µL/min was used to elute peptides directly into a Waters Synapt G2-Si mass spectrometer (Waters, Milford, MA). HDMSE data were acquired with an IMS wave velocity of 600 m/s. A 20 to 30 V ramp trap CE was used to facilitate high-energy acquisition of product ions, and continuous lock mass (Leu-Enk) was acquired for mass accuracy. RSVpol peptides were identified using ProteinLynx Global Server 3.0.3 and further filtered using DynamX 3.0 (Waters). A LEAP autosampler controlled by Chronos software was used for all apo and ligand-bound experiments. HDX reactions (Buffer B prepared in D_2_O) were performed at 25°C and quenched at various times (10, 100, and 1,000 s) with 50 µL of ice-cold quench buffer. All deuteration time points were acquired in duplicate. HDX-MS analysis was performed using DynamX 3.0, and deuterium uptake difference plots, ΔDt (Apo–Ligand bound), displaying the difference in percent deuteration between apo and ligand states were generated. Statistically significant differences in deuterium uptake between pair-wise states were identified using 95% confidence intervals for the ΔDt plots.

### Metabolic stability in liver microsomes

Test compounds originating from 10 mM DMSO solution were incubated under the generic condition (0.5 mg/mL microsomal protein, 1 mM NADPH, 1 mM MgCl_2_, and 0.1 M phosphate buffer, pH 7.4, 37°C) at a defined substrate concentration (typically 1 µM) with human liver microsomes across a time course (typically 0, 10, and 60 min). Liver microsomes, buffer, and test compound were pre-incubated for 5 min. The cofactor NADPH was added to initiate the reaction, and the reaction was terminated by the addition of acetonitrile. The samples were centrifuged prior to analysis by liquid chromatography-tandem mass spectrometry. The relative amount of parent compound remaining in the active incubations versus the control incubations (t = 0 min) for each compound was measured by peak area comparison. The *in vitro* metabolic half-life (t_1/2_) was calculated using the slope (k) of the log-linear regression from the percentage parent compound remaining versus time relationship. The *in vitro* CL_int_ (µL/min/mg microsomal protein) was calculated using the *in vitro* metabolic t_1/2_, incubation volume, and the weight of microsomal protein in the incubation.

### Chemistry

**Fig 6:**
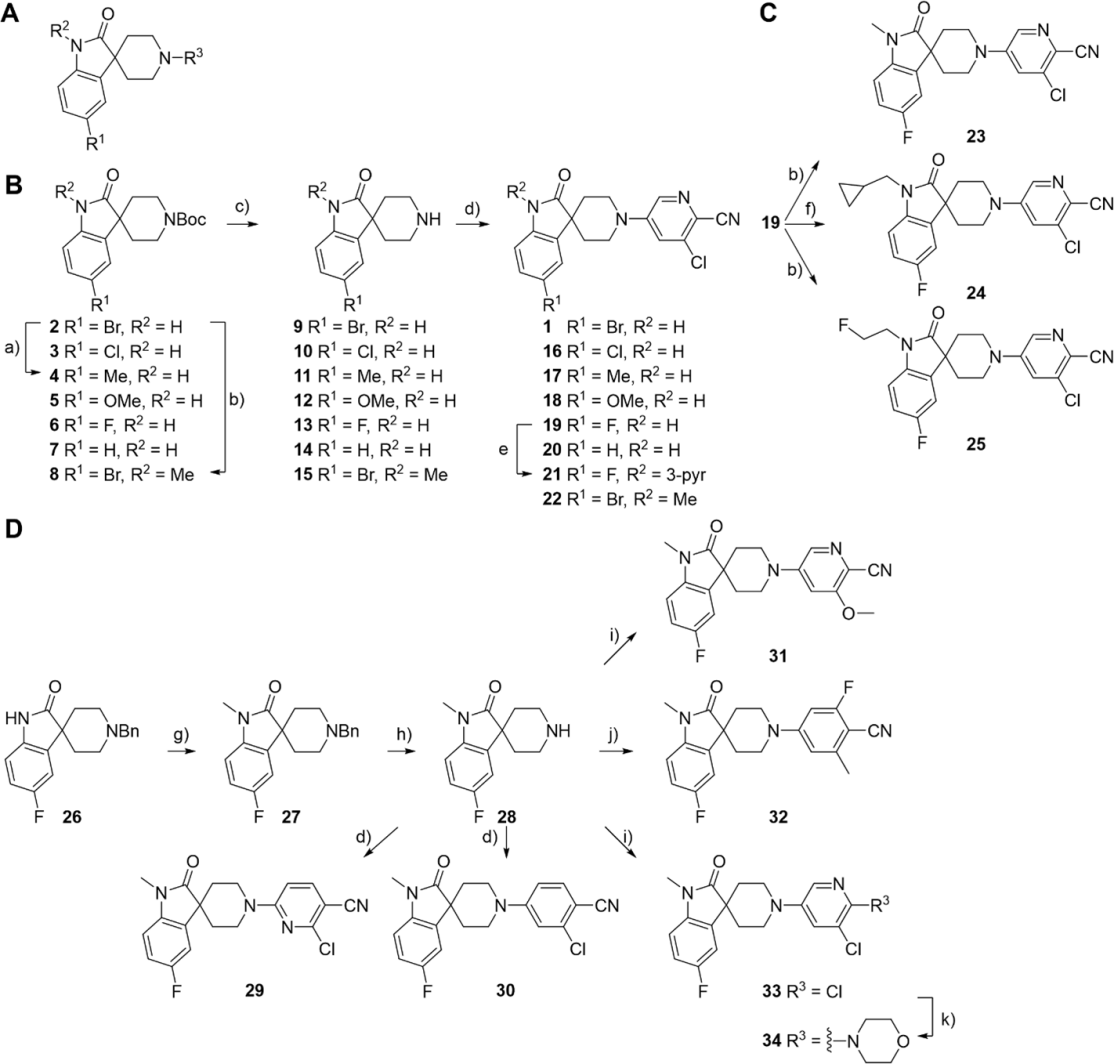
Chemical synthesis of **1** and its derivatives. (**A**) The three vectors being explored: southern R^1^, left-hand-side R^2^, and right-hand-side R^3^. (**B**) Chemical syntheses of **1** and **18**–**22**. Conditions: (a) trimethylboroxine, Pd(dppf)Cl_2_, K_2_CO_3_, 1,4-dioxane, water, 100°C; (b) alkyliodide, NaH, DMF, rt; (c) HCl, 1,4-dioxane, 100°C; (d) ArX, DIPEA, NMP, 80°C; (e) 3-bromopyridine, DMEDA, CuI, Cs_2_CO_3_, toluene, DMF, 100°C. (**C**) Chemical syntheses of **23**–**25**. Conditions: (f) DIAD, cyclopropane methanol, P(*t*-Bu)_3_, THF, 0°C to rt. (**D**) Chemical syntheses of **29**–**32** and **34**. Conditions: (g) MeI, Cs_2_CO_3_, DMF, 0°C; (h) ACE-Cl, DIPEA, CH_3_CN, toluene, 60°C; (i) ArX, NaO*t*-Bu, RuPhos Pd G4, 1,4-dioxane; (j) 4-bromo-2-fluoro-6-methylbenzonitrile, NaOTMS, RuPhos Pd G4, 1,4-dioxane, 80°C; (k) morpholine, RuPhos Pd G4, NaO*t*-Bu, 1,4-dioxane, 65°C. X, appropriate halide; DMF, dimethylformamide DIPEA, N,N-diisopropylethylamine; NMP, N-methylpyrrolidone; DMEDA, N,N-dimethylethylenediamine; DIAD, diisopropyl azodicarboxylate; ACE-Cl, 1-chloroethyl chloroformate; RuPhos Pd G4, [dicyclohexyl(2′,6′-diisopropoxy-2-biphenylyl)phosphine-κ*P*](methanesulfonatato-κ*O*)[2′-(methylamino-κ*N*)-2-biphenylyl-κC^2^]palladium.

Synthetic routes are depicted in Fig. 6. Detailed synthetic procedures are described in the Supporting information section “Synthesis.” Nuclear magnetic resonance (NMR) spectra were recorded on a Bruker Avance DRX 400 spectrometer or a Bruker Avance III 400 spectrometer. Chemical shifts were reported in parts per million (ppm) relative to TMS (δ = 0 ppm) and coupling constant(s) *J* in Hertz (Hz). The high-resolution mass spectrometry (HRMS) experiments were performed in full MS scan type mode on a Q-Exactive mass spectrometer via an electrospray ionization (ESI) interface, and the reported accurate masses correspond to the [M+H]^+^ (protonated monoisotopic molecular mass). Liquid chromatography mass spectrometry (LC-MS) was recorded on Waters:Acquity UPLC-DAD and SQD or SQD2 instruments, and compounds are described by their experimental retention times (Rt) and ions. Full descriptions of all analytical methods are available in the Supporting information section “HPLC and LC-MS.” The ^1^H NMR and mass spectrometry data for **1**, **22,** and analogs **16** to **21**, **23** to **25**, **29** to **32** and **34** are provided herewith. ^13^C NMR and HRMS data for **1** and **22** are also provided. Compound **1** (5-(5-bromo-2-oxospiro[indoline-3,4'-piperidin]-1'-yl)-3-chloropicolinonitrile) ^1^H NMR (400 MHz, DMSO-d_6_) δ ppm 1.70–1.98 (m, 4 H) 3.77–3.97 (m, 4 H) 6.82 (d, *J* = 8.1 Hz, 1 H) 7.37 (dd, *J* = 8.3, 2.1 Hz, 1 H) 7.60 (d, *J* = 2.6 Hz, 1 H) 7.70 (d, *J* = 2.0 Hz, 1 H) 8.43 (d, *J* = 2.6 Hz, 1 H) 10.58 (s, 1 H); ^13^C NMR (101 MHz, DMSO-d_6_) δ ppm 30.94 (2 C) 41.59 (2 C) 44.83 (1 C) 111.28 (1 C) 113.44 (1 C) 116.40 (1 C) 116.51 (1 C) 118.54 (1 C) 126.48 (1 C) 130.58 (1 C) 135.44 (1 C) 136.13 (1 C) 136.45 (1 C) 140.38 (1 C) 148.39 (1 C) 180.26 (1 C); LCMS: Rt: 1.94 min, 95%, 417 [M+H]^+^, Method A; HRMS (ESI+) calculated for C_18_H_15_BrClN_4_O [M+H]^+^: 417.01123, found: 417.01035. Compound **22** (5-(5-bromo-1-methyl-2-oxospiro[indoline-3,4'-piperidin]-1'-yl)-3-chloropicolinonitrile) ^1^H NMR (400 MHz, DMSO-d_6_) δ ppm 1.73–1.84 (m, 2 H) 1.88–1.99 (m, 2 H) 1.94 (s, 1 H) 3.13 (s, 3 H) 3.81–3.96 (m, 4 H) 6.98–7.04 (m, 2 H) 7.00 (s, 1 H) 7.46–7.53 (m, 1 H) 7.58–7.64 (m, 1 H) 7.75 (d, *J* = 1.9 Hz, 1 H) 8.44 (d, *J* = 2.6 Hz, 1 H); ^13^C NMR (101 MHz, DMSO-d_6_) δ ppm 26.51 (1 C) 31.46 (2 C) 42.13 (2 C) 45.00 (1 C) 110.91 (1 C) 114.68 (1 C) 116.94 (1 C) 116.99 (1 C) 119.05 (1 C) 126.65 (1 C) 131.13 (1 C) 135.94 (1 C) 136.08 (1 C) 136.63 (1 C) 142.46 (1 C) 148.87 (1 C) 178.71 (1 C); LCMS: Rt: 1.14 min, 100%, 433 [M+H]^+^, Method B; HRMS (ESI+) calculated for C_19_H_17_BrClN_4_O [M+H]^+^: 431.02688, found: 431.02586. Compound **16** (3-chloro-5-(5-chloro-2-oxospiro[indoline-3,4'-piperidin]-1'-yl)picolinonitrile) ^1^H NMR (400 MHz, DMSO-d_6_) δ ppm 1.64–2.06 (m, 4 H) 3.87 (br t, *J* = 4.1 Hz, 4 H) 6.86 (d, *J* = 8.4 Hz, 1 H) 7.24 (dd, *J* = 8.3, 2.1 Hz, 1 H) 7.59 (dd, *J* = 3.4, 2.5 Hz, 2 H) 8.42 (d, *J* = 2.6 Hz, 1 H) 10.57 (s, 1 H); LCMS: Rt: 1.07 min, 99%, 373 [M+H]^+^, Method F. Compound **17** (3-chloro-5-(5-methyl-2-oxospiro[indoline-3,4'-piperidin]-1'-yl)picolinonitrile) ^1^H NMR (400 MHz, DMSO-d_6_) δ ppm 1.81 (t, *J* = 5.6 Hz, 4 H) 2.25 (s, 3 H) 3.78–3.98 (m, 4 H) 6.75 (d, *J* = 7.7 Hz, 1 H) 7.00 (m, *J* = 7.9 Hz, 1 H) 7.29 (s, 1 H) 7.62 (d, *J* = 2.4 Hz, 1 H) 8.44 (d, *J* = 2.6 Hz, 1 H) 10.34 (s, 1 H); LCMS: Rt: 2.31 min, 100%, 353 [M+H]^+^, Method J. Compound **18** (3-chloro-5-(5-methoxy-2-oxospiro[indoline-3,4'-piperidin]-1'-yl)picolinonitrile) ^1^H NMR (400 MHz, DMSO-d_6_) δ ppm 1.75–1.93 (m, 4 H) 3.70 (s, 3 H) 3.81–3.94 (m, 4 H) 6.74–6.79 (m, 2 H) 7.06–7.11 (m, 1 H) 7.60 (d, *J* = 2.6 Hz, 1 H) 8.43 (d, *J* = 2.6 Hz, 1 H) 10.26 (s, 1 H); LCMS: Rt: 0.92 min, 100%, 369 [M+H]^+^, Method E. Compound **19** (3-chloro-5-(5-fluoro-2-oxospiro[indoline-3,4'-piperidin]-1'-yl)picolinonitrile) ^1^H NMR (400 MHz, DMSO-d_6_) δ ppm 1.78–1.92 (m, 4 H) 3.80–3.96 (m, 4 H) 6.85 (dd, *J* = 8.5, 4.5 Hz, 1 H) 6.95–7.10 (m, 1 H) 7.45 (dd, *J* = 8.9, 2.5 Hz, 1 H) 7.61 (d, *J* = 2.6 Hz, 1 H) 8.44 (d, *J* = 2.4 Hz, 1 H) 10.47 (s, 1 H); LCMS: Rt: 1.83 min, 97%, 357 [M+H]^+^, Method A. Compound **22** (3-chloro-5-(2-oxospiro[indoline-3,4'-piperidin]-1'-yl)picolinonitrile) ^1^H NMR (400 MHz, DMSO-d_6_) δ ppm 1.83 (t, *J* = 5.7 Hz, 4 H) 3.75–4.02 (m, 4 H) 3.89–3.94 (m, 1 H) 6.87 (d, *J* = 7.7 Hz, 1 H) 6.95 (br d, *J* = 0.9 Hz, 1 H) 7.20 (td, *J* = 7.7, 1.1 Hz, 1 H) 7.45 (d, *J* = 7.0 Hz, 1 H) 7.62 (d, *J* = 2.6 Hz, 1 H) 8.44 (d, *J* = 2.6 Hz, 1 H) 10.39–10.53 (m, 1 H); LCMS: Rt: 1.79 min, 100.00, 339.2 [M+H]^+^, Method I. Compound **21** (3-chloro-5-(5-fluoro-2-oxo-1-(pyridin-3-yl)spiro[indoline-3,4'-piperidin]-1'-yl)picolinonitrile) ^1^H NMR (400 MHz, DMSO-d_6_) δ ppm 1.97–2.12 (m, 4 H) 3.79–4.05 (m, 4 H) 6.73–6.83 (m, 1 H) 7.01–7.13 (m, 1 H) 7.52–7.69 (m, 3 H) 7.86–8.06 (m, 1 H) 8.42–8.50 (m, 1 H) 8.64–8.74 (m, 2 H); LCMS: Rt: 1.90 min, 100%, 434 [M+H]^+^, Method C. Compound **23** (3-chloro-5-(5-fluoro-1-methyl-2-oxospiro[indoline-3,4'-piperidin]-1'-yl)picolinonitrile) ^1^H NMR (400 MHz, DMSO-d_6_) δ ppm 1.66–2.06 (m, 4 H) 3.14 (s, 3 H) 3.70–4.08 (m, 4 H) 6.96–7.08 (m, 1 H) 7.09–7.22 (m, 1 H) 7.52 (dd, *J* = 8.7, 2.5 Hz, 1 H) 7.62 (d, *J* = 2.6 Hz, 1 H) 8.44 (d, *J* = 2.6 Hz, 1 H); LCMS: Rt: 1.98 min, 97%, 371 [M+H]^+^, Method D. Compound **24** (3-chloro-5-(1-(cyclopropylmethyl)-5-fluoro-2-oxospiro[indoline-3,4'-piperidin]-1'-yl)picolinonitrile) ^1^H NMR (400 MHz, DMSO-d_6_) δ ppm 0.28–0.35 (m, 2 H) 0.40–0.50 (m, 2 H) 1.06–1.19 (m, 1 H) 1.71–1.98 (m, 4 H) 3.58 (d, *J =* 6.9 Hz, 2 H) 3.90 (t, *J =* 5.7 Hz, 4 H) 7.06–7.20 (m, 2 H) 7.53 (dd, *J =* 8.7, 2.4 Hz, 1 H) 7.62 (d, *J =* 2.6 Hz, 1 H) 8.44 (d, *J =* 2.6 Hz, 1 H); LCMS: Rt: 2.15 min, 100%, 411 [M+H]^+^, Method C. Compound **25** (3-chloro-5-(5-fluoro-1-(2-fluoroethyl)-2-oxospiro[indoline-3,4'-piperidin]-1'-yl)picolinonitrile) ^1^H NMR (400 MHz, DMSO-d_6_) δ ppm 1.67–1.99 (m, 3 H) 1.87–1.96 (m, 1 H) 3.89 (br s, 4 H) 4.02 (br d, *J* = 26.7 Hz, 2 H) 4.63 (br d, *J* = 47.3 Hz, 2 H) 7.12 (d, *J* = 5.9 Hz, 2 H) 7.48–7.54 (m, 1 H) 7.59–7.64 (m, 1 H) 8.44 (d, *J* = 2.6 Hz, 1 H); LCMS: Rt: 1.07 min, 97%, 403 [M+H]^+^, Method B. Compound **29** (2-chloro-6-(5-fluoro-1-methyl-2-oxospiro[indoline-3,4'-piperidin]-1'-yl)nicotinonitrile) ^1^H NMR (400 MHz, DMSO-d_6_) δ ppm 1.68–1.98 (m, 4 H) 3.14 (s, 3 H) 3.93–4.17 (m, 4 H) 6.97–7.07 (m, 2 H) 7.10–7.18 (m, 1 H) 7.47–7.56 (m, 1 H) 7.95 (d, *J* = 8.9 Hz, 1 H); LCMS: Rt: 1.12 min, 100%, 371 [M+H]^+^, Method B. Compound **30** (4-(5-fluoro-1-methyl-2-oxospiro[indoline-3,4'-piperidin]−1'-yl)-2-methylbenzonitrile) ^1^H NMR (400 MHz, DMSO-d_6_) δ ppm 1.73–1.93 (m, 4 H) 3.14 (s, 3 H) 3.70–3.90 (m, 4 H) 7.00–7.08 (m, 2 H) 7.10–7.18 (m, 1 H) 7.20–7.23 (m, 1 H) 7.47–7.54 (m, 1 H) 7.64–7.69 (m, 1 H); LCMS: Rt: 2.10 min, 100%, 370 [M+H]^+^, Method A. Compound **31** (5-(5-fluoro-1-methyl-2-oxospiro[indoline-3,4'-piperidin]-1'-yl)-3-methoxypicolinonitrile) ^1^H NMR (400 MHz, DMSO-d_6_) δ ppm 1.78–1.96 (m, 4 H) 3.14 (s, 3 H) 3.80–3.91 (m, 4 H) 3.91–3.96 (m, 3 H) 6.99–7.02 (m, 1 H) 7.02–7.07 (m, 1 H) 7.10–7.19 (m, 1 H) 7.51 (dd, *J* = 8.7, 2.5 Hz, 1 H) 8.08 (d, *J* = 2.3 Hz, 1 H); LCMS: Rt: 1.81 min, 100%, 367 [M+H]^+^, Method A. Compound **32** (2-fluoro-4-(5-fluoro-1-methyl-2-oxospiro[indoline-3,4'-piperidin]-1'-yl)-6-methylbenzonitrile) ^1^H (600 MHz, DMSO-d_6_) δ ppm 1.81 (br d, *J* = 4.40 Hz, 4 H) 2.39 (s, 3 H) 3.13 (s, 3 H) 3.49–3.52 (m, 83 H) 3.72–3.79 (m, 2 H) 3.79–3.87 (m, 2 H) 6.57–6.57 (m, 1 H) 6.86 (s, 2 H) 7.00–7.07 (m, 1 H) 7.09–7.21 (m, 1 H) 7.39–7.54 (m, 1 H); LCMS: Rt: 1.14 min, 100%, 368 [M+H]^+^, Method G. Compound **34** (1'-(5-chloro-6-morpholinopyridin-3-yl)-5-fluoro-1-methylspiro[indoline-3,4'-piperidin]-2-one) ^1^H NMR (400 MHz, DMSO-d_6_) δ ppm 1.87 (t, *J* = 5.6 Hz, 4 H) 3.06–3.11 (m, 4 H) 3.13 (s, 3 H) 3.41–3.50 (m, 2 H) 3.53–3.62 (m, 2 H) 3.71–3.77 (m, 4 H) 7.00–7.06 (m, 1 H) 7.09–7.18 (m, 1 H) 7.50 (dd, *J* = 8.7, 2.5 Hz, 1 H) 7.56 (d, *J* = 2.6 Hz, 1 H) 8.05 (d, *J =* 2.9 Hz, 1 H); LCMS: Rt: 2.05 min, 99%, 431 [M+H]^+^, Method C.

## Data Availability

The cryoEM structure of compound **22** bound to RSV L+P has been deposited in the Electron Microscopy Data Bank (EMDB) under the accession ID EMD-48846 and in the Protein Data Bank (PDB) under PDB ID 9N36.

## SUPPLEMENTAL MATERIAL

Supplemental material jvi.00178-25-s0001.pdf Figures S1 to S10, Tables S1 and S2, equations, and chemistry procedures.

ASM does not own the copyrights to Supplemental Material that may be linked to, or accessed through, an article. The authors have granted ASM a non-exclusive, world-wide license to publish the Supplemental Material files. Please contact the corresponding author directly for reuse.
