# Supplementary material for: Discovery of a non-nucleoside inhibitor that binds to a novel site in the palm domain of the respiratory syncytial virus RNA-dependent RNA polymerase
Source: J Virol. 2025 Jun 2;99(7):e00178-25. doi: 10.1128/jvi.00178-25 (PMC12282092; doi:10.1128/jvi.00178-25)
Supplement: Supplemental material — Figures S1 to S10, Tables S1 and S2, equations, and chemistry procedures. [file jvi.00178-25-s0001.pdf]

Discovery of a Non-Nucleoside Inhibitor that Binds to a Novel site in the Palm Domain of the Respiratory Syncytial Virus RNA-dependent RNA polymerase

Jay H. Kalin,[1#*] Yanting Yin,[1#*] Minh T. Tran,[2#] Madison Piassek,[1] Amy Fung,[3] Sandrine Grosse,[2] Edgar Jacoby,[2] Anusarka Bhaumik,[1] Suraj Adhikary,[1] Robyn Miller,[1] Cynthia Lemmens,[2] Ferdinand H. Lutter,[2] Serge Pieters,[2] Ludwig Cooymans,[2] Geert Rombouts,[2] Daniel Oehlrich,[2] Sonia Tomaso,[3] Kate Lozada,[3] Miguel Osorio Garcia,[3] Brandon Anson,[3] Suzanne De Bruyn,[2] Constance Smith-Monroy,[1] Jean-Marc Neefs,[2] Nádia Conceição-Neto,[2] Bart Stoops,[2] Herman van Vlijmen,[2] Aaron Patrick,[1] Xiaodi Yu,[1] Victoria Wong,[1] Daniel Krosky,[1] Pravien Abeywickrema,[1] Stephen Mason,[3] Zhinan Jin,[3] Tim H.M. Jonckers,[2] Sujata Sharma[1]

[1]Janssen Research & Development, LLC, a Johnson & Johnson Company, Spring House, Pennsylvania, USA
[2]Janssen Pharmaceutica N.V., Beerse, Belgium
[3]Janssen Research & Development, LLC, a Johnson & Johnson Company, Brisbane, California, USA
[#]Authors contributed equally
*Corresponding author

Supplementary Information

Table of Contents

1. Supplementary Figures……………………………………………………………………S2
2. Supplementary Tables…………………………………………………………………..S12
3. Supplementary Equations…………………………………………………………………S14
4. Experimental Procedures for Chemistry……………………………………………..S15

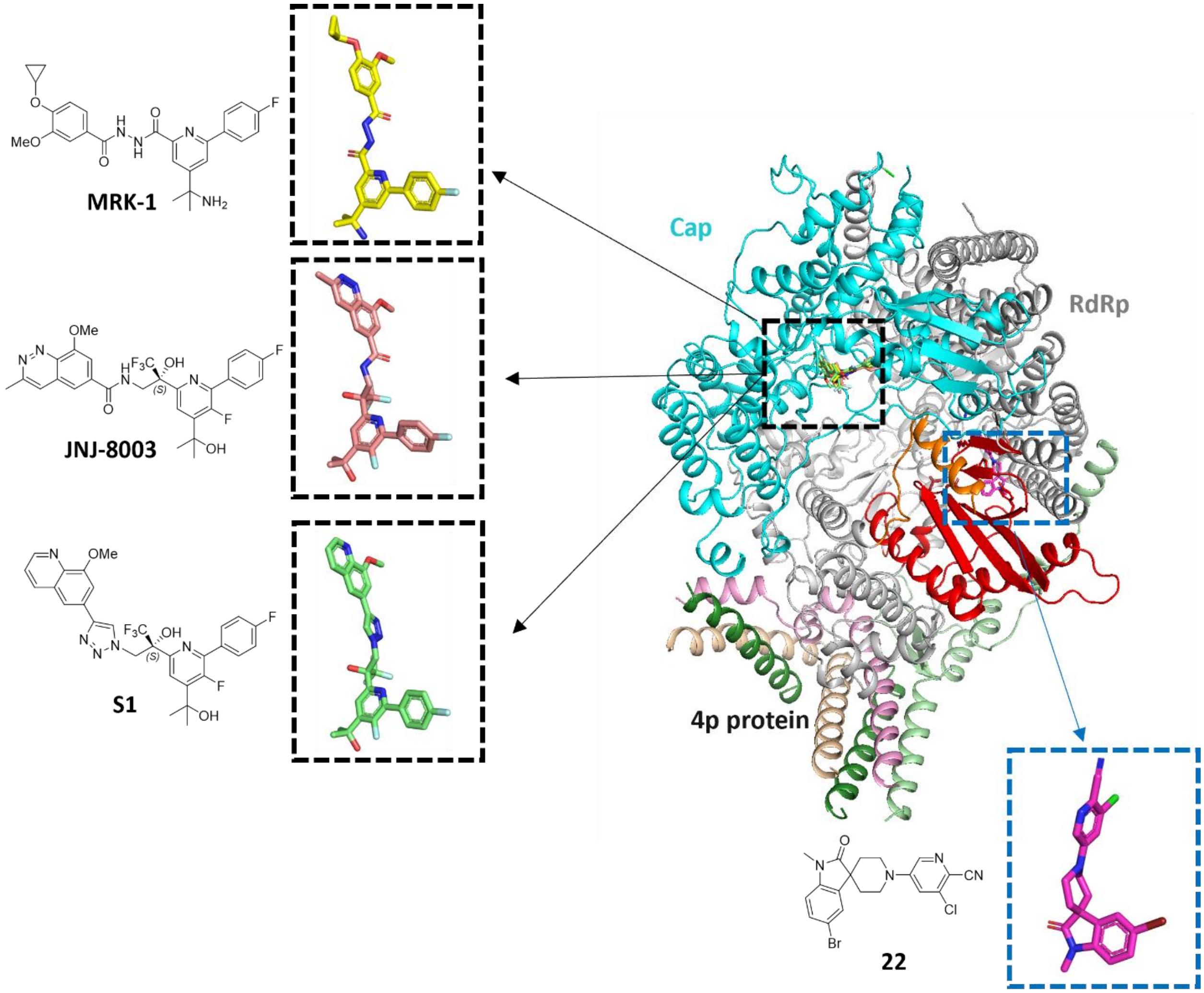

SUPP FIG S1 Cryo-EM structures of RSV L+P in complex with MRK-1, JNJ-8003, S1, and compound **22**. The RdRp domain, capping domain, palm domain, and 4 partial P proteins are color-coded in gray, cyan, red, and light green, pink, light orange, green, respectively. The zoomed-in view highlights MRK-1, JNJ-8003, **S1**, and compound **22**, which are labeled in yellow, pink, green, and magenta, respectively.

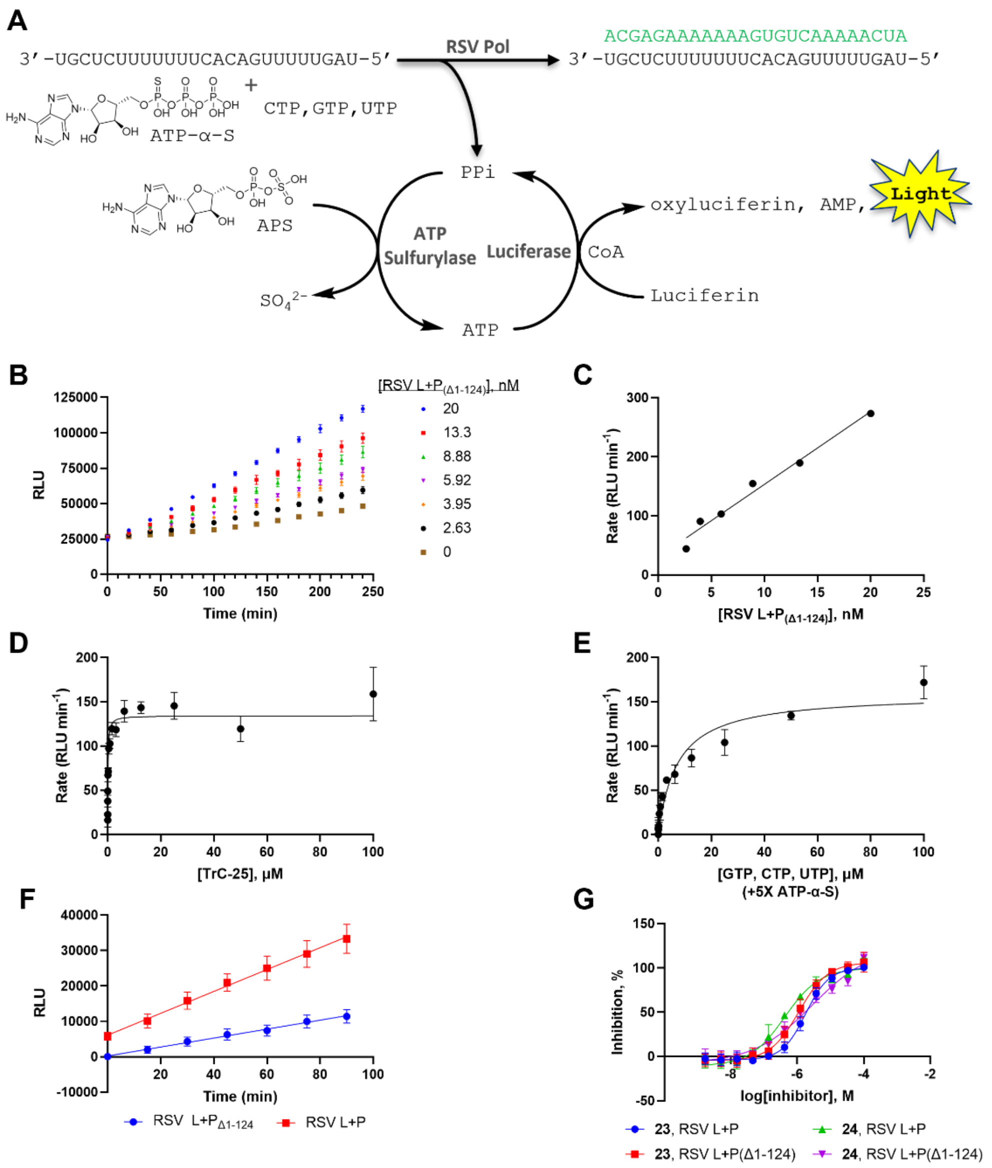

SUPP FIG S2 Kinetic characterization of truncated RSV L+P$_{(\Delta 1\text{-}124)}$ screening construct. (A) Assay schematic illustrating coupling of pyrophosphate production to luminescent signal. (B) Reaction progress curves generated for 3 – 20 nM RSV L+P$_{(\Delta 1\text{-}124)}$ (*n = 4*). (C) Background subtracted initial rates determined from the linear portion of each curve were plotted against enzyme concentration to establish a linear range (*n = 4*). (D) Steady-state kinetics with TrC-25 RNA template and (E) pooled nucleotide substrates. Background subtracted initial rates extracted from progress curves were plotted against substrate concentration and fit to the Michaelis-Menten equation (*n = 2*). (F) Background subtracted reaction progress curves comparing the truncated and full-length constructs. 50 nM enzyme, 10 μM ATP/GTP/CTP, 100 μM ATPαS, 5 μM TrC-25. $\nu_{(\text{RSV L+P}(\Delta 1\text{-}124))}$ = 126 RLU min^{-1}, $\nu_{(\text{RSV L+P})}$ = 309 RLU min^{-1} (*n = 4*). (G) Dose-response curves comparing potency of tool compounds against full-length RSV L+P and the truncated screening construct RSV L+P$_{(\Delta 1\text{-}124)}$. **23**, RSV L+P IC_{50} = 1.78 μM, RSV L+P$_{(\Delta 1\text{-}124)}$ IC_{50} = 1.09 μM. **24**, RSV L+P IC_{50} = 0.42 μM, RSV L+P$_{(\Delta 1\text{-}124)}$ IC_{50} = 1.53 μM (*n* ≥ *4*). Data are representative of independent experiments and plotted as mean ± SD.

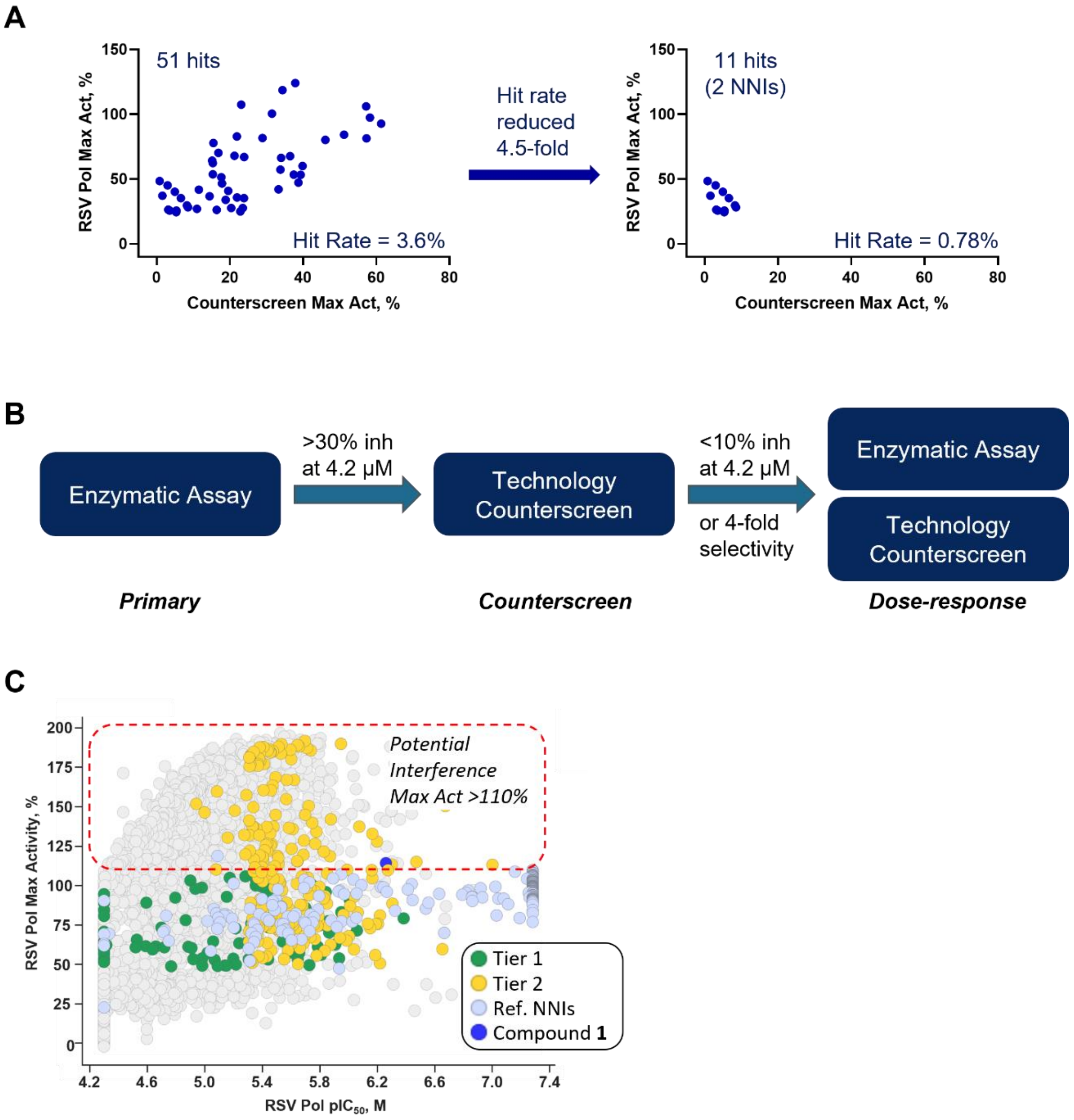

SUPP FIG S3 Technology counterscreen and HTS strategy. (A) False positives could be filtered out by screening against the coupled enzyme system. (B) Progression of chemical matter. Compounds were screened in single-point at 4.2 µM. Hits showing > 30% inhibition were tested in duplicate against the coupled enzyme system. Hits showing < 10% inhibition in the counterscreen or ≥ 4-fold more activity in the primary assay were progressed to dose-response and run in duplicate in both assays. (C) Inhibitor potency and maximum percent inhibition were compared to a set of reference NNIs to establish a desired pharmacological profile. Tier 1 hits (88 compounds) were well-correlated with reference NNIs and displayed minimal activity in the pyrophosphate detection counterscreen. Tier 2 hits (185 compounds) displayed a range of activities in the counterscreen but were still active at lower concentrations in the primary assay. Compound **1** was selected for further investigation due to its drug-like properties, structural novelty, and confirmation of biochemical activity after resynthesis.

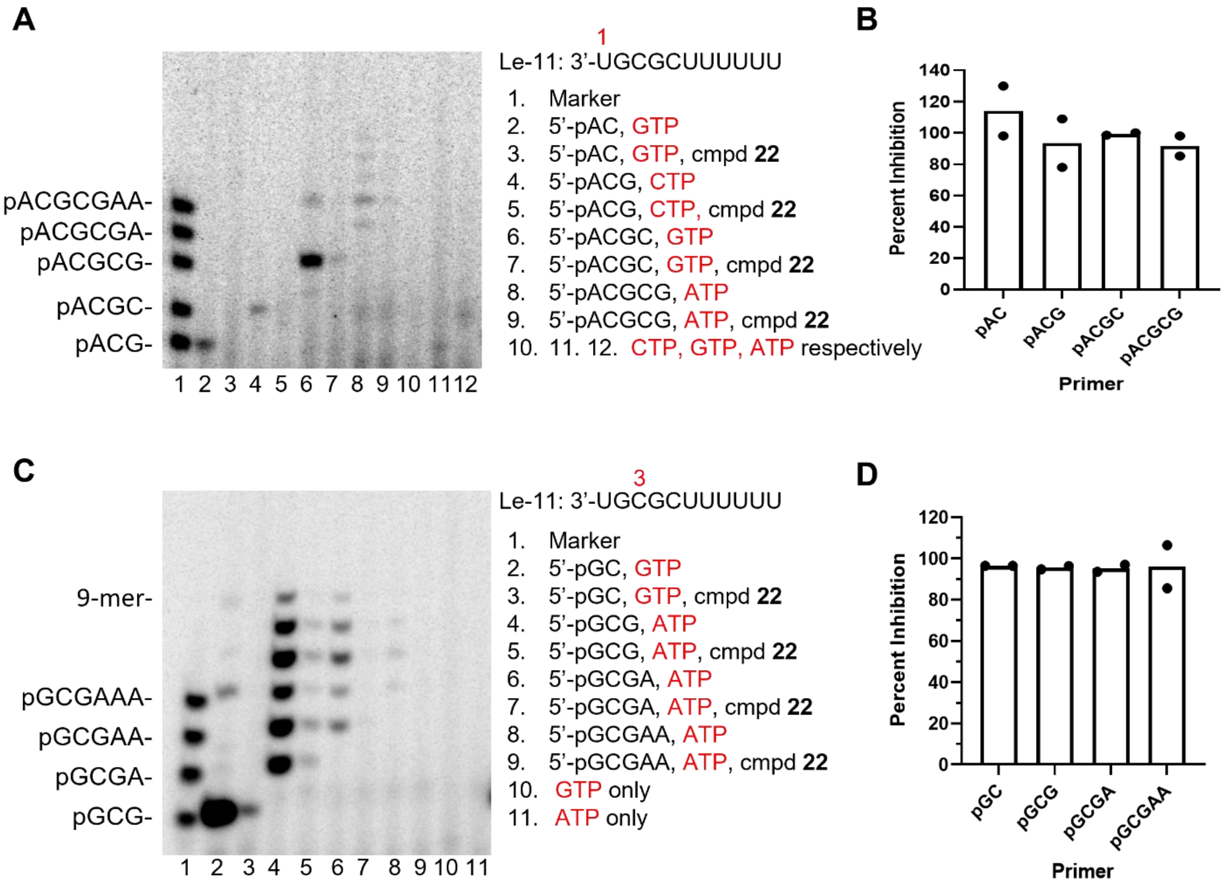

SUPP FIG S4 Compound **22** inhibits elongation of viral genome and RNA transcripts.
(A) Polyacrylamide gel showing single nucleotide incorporation (SNI) from a set of short primers with or without 50 µM compound **22** with Le-11 template (+1 site RNA synthesis). (B) Bars indicate the mean, and the data points from two independent experiments are shown. (C) Gel image showing SNI from a set of short primers with or without 50 µM compound **22** with Le-11 template (+3 site RNA synthesis). (D) Bars indicate the mean, and the data points from two independent experiments are shown.

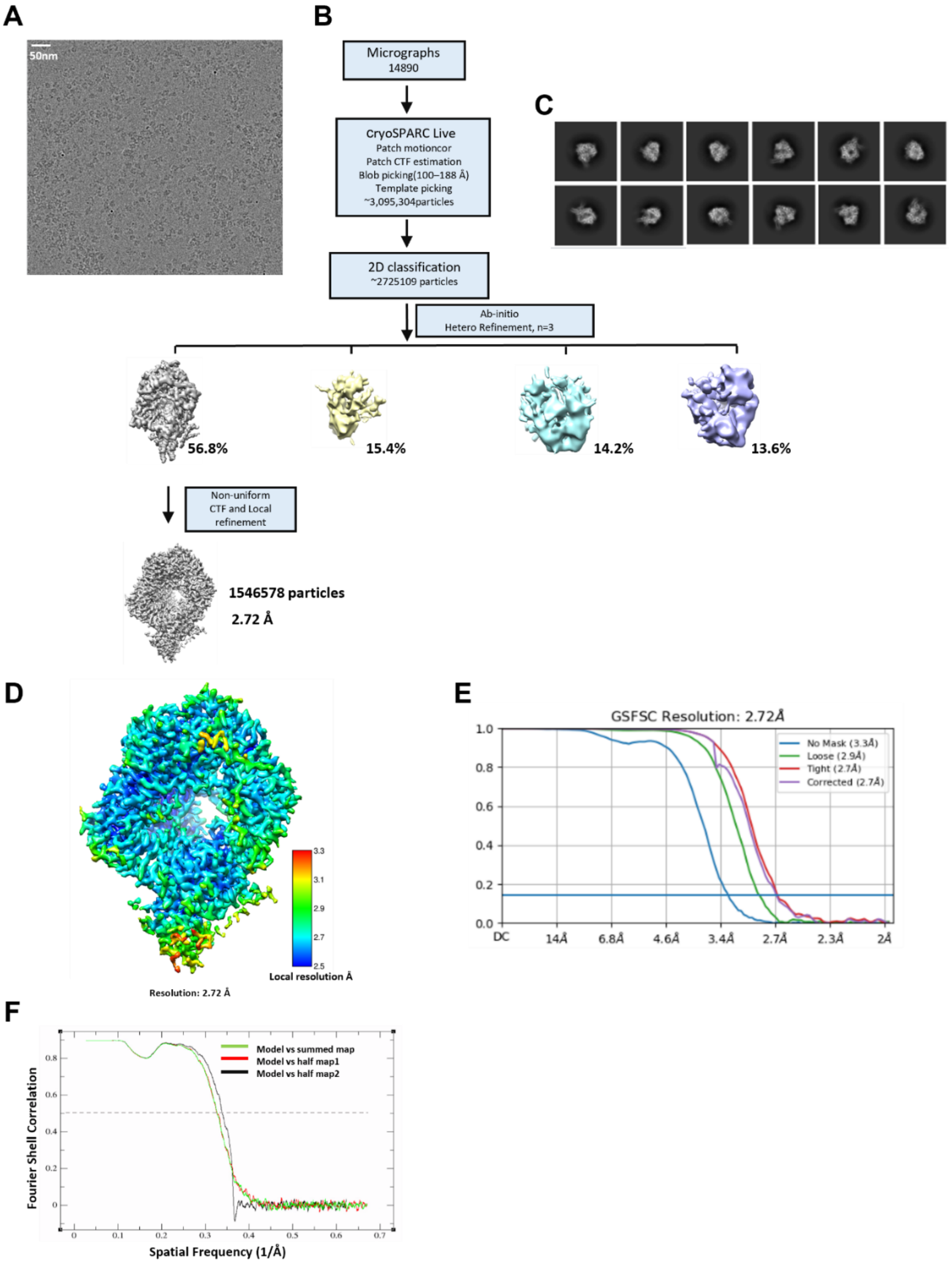

SUPP FIG S5 CryoEM Analysis of RSVpol-compound **22**. (A) Micrograph from RSVpol-compound **22** data collection. (B) Workflow: CryoEM data analysis for RSVpol-compound **22** map. CryoSPARC Live was used for real-time data processing. The non-uniform analysis yielded a 2.72 Å resolution 3D map. (C) Representative 2D class averages of RSVpol-compound **22**. (D) Local resolution colored by ResMap estimation. (E) Average resolutions estimated using 0.143 criterion of gold standard Fourier shell correlation (GSFSC). (F) Model validation through FSC curve comparisons: model vs. half map 1 (work), model vs. half map 2 (free), model vs. full map in red, black, and green.

Country	sequence	aligned_da	336	337	340	356	363	423	698	699	700	812	857	858	859	860	861	868	871	873	885
CONSENSUS			E	G	M	F	N	Y	I	T	D	N	G	T	E	T	Y	F	K	I	K
2nd most frequent aa																					
3rd most frequent aa																					
	Subtype	A	E	G	M	F	N	Y	I	T	D	N	G	T	E	T	Y	F	K	I	K
		B	E	G	M	F	N	Y	I	T	D	N	G	T	E	T	Y	F	K	I	K
	Overall		E	G	M	F	N	Y	I	T	D	N	G	T	E	T	Y	F	K	I	K
	Differences																				

Difference from Reference Seq by time (counts)

RSVA

REFERENCE RSVA : MW039343	E	G	M	F	N	Y	I	T	D	N	G	T	E	T	Y	F	K	I	K
# Sequences different than Reference RSVA - ever	3	4	3	1	3	0	0	0	0	0	1	1	1	0	0	1	1	1	1
until 1980	0	0	0	0	0	0	0	0	0	0	0	0	0	0	0	0	0	0	0
1981-1990	0	0	0	0	0	0	0	0	0	0	0	0	0	0	0	0	0	0	0
1991-2000	0	0	0	0	0	0	0	0	0	0	0	0	0	0	0	0	0	0	0
2001-2010	0	0	0	0	0	0	0	0	0	0	0	0	0	0	0	0	0	0	0
2011-2020	3	4	3	1	3	0	0	0	0	0	1	1	1	0	0	1	1	1	1
after 2020	0	0	0	0	0	0	0	0	0	0	0	0	0	0	0	0	0	0	0

high counts = many changes

Difference from Reference Seq by time (percent frequency)

RSVA

REFERENCE RSVA : JX198112	E	G	M	F	N	Y	I	T	D	N	G	T	E	T	Y	F	K	I	K
**# Sequences different than Reference RSVA - ever**	0.1%	0.1%	0.1%	0.0%	0.1%	0.0%	0.0%	0.0%	0.0%	0.0%	0.0%	0.0%	0.0%	0.0%	0.0%	0.0%	0.0%	0.0%	0.0%
until 1980	0.0%	0.0%	0.0%	0.0%	0.0%	0.0%	0.0%	0.0%	0.0%	0.0%	0.0%	0.0%	0.0%	0.0%	0.0%	0.0%	0.0%	0.0%	0.0%
1981-1990	0.0%	0.0%	0.0%	0.0%	0.0%	0.0%	0.0%	0.0%	0.0%	0.0%	0.0%	0.0%	0.0%	0.0%	0.0%	0.0%	0.0%	0.0%	0.0%
1991-2000	0.0%	0.0%	0.0%	0.0%	0.0%	0.0%	0.0%	0.0%	0.0%	0.0%	0.0%	0.0%	0.0%	0.0%	0.0%	0.0%	0.0%	0.0%	0.0%
2001-2010	0.0%	0.0%	0.0%	0.0%	0.0%	0.0%	0.0%	0.0%	0.0%	0.0%	0.0%	0.0%	0.0%	0.0%	0.0%	0.0%	0.0%	0.0%	0.0%
2011-2020	0.2%	0.2%	0.2%	0.1%	0.2%	0.0%	0.0%	0.0%	0.0%	0.0%	0.1%	0.1%	0.1%	0.0%	0.0%	0.1%	0.1%	0.1%	0.1%
after 2020	0.0%	0.0%	0.0%	0.0%	0.0%	0.0%	0.0%	0.0%	0.0%	0.0%	0.0%	0.0%	0.0%	0.0%	0.0%	0.0%	0.0%	0.0%	0.0%

high counts = many changes

Difference from Reference Seq by time (counts)

RSVB

REFERENCE RSVB : KU316116	E	G	M	F	N	Y	I	T	D	N	G	T	E	T	Y	F	K	I	K
**# Sequences different than Reference RSVB - ever**	3	4	3	4	3	1	0	0	0	0	0	3	0	0	0	0	1	0	0
until 1980	0	0	0	0	0	0	0	0	0	0	0	0	0	0	0	0	0	0	0
1981-1990	0	0	0	0	0	0	0	0	0	0	0	0	0	0	0	0	0	0	0
1991-2000	0	0	0	0	0	0	0	0	0	0	0	0	0	0	0	0	0	0	0
2001-2010	0	0	0	0	0	0	0	0	0	0	0	0	0	0	0	0	0	0	0
2011-2020	3	3	3	4	3	1	0	0	0	0	0	3	0	0	0	0	1	0	0
after 2020	0	1	0	0	0	0	0	0	0	0	0	0	0	0	0	0	0	0	0

high counts = many changes

Difference from Reference Seq by time (percent frequency)

RSVB

REFERENCE RSVB : KU316116	E	G	M	F	N	Y	I	T	D	N	G	T	E	T	Y	F	K	I	K
**# Sequences different than Reference RSVB - ever**	0.1%	0.2%	0.1%	0.2%	0.1%	0.0%	0.0%	0.0%	0.0%	0.0%	0.0%	0.1%	0.0%	0.0%	0.0%	0.0%	0.0%	0.0%	0.0%
until 1980	0.0%	0.0%	0.0%	0.0%	0.0%	0.0%	0.0%	0.0%	0.0%	0.0%	0.0%	0.0%	0.0%	0.0%	0.0%	0.0%	0.0%	0.0%	0.0%
1981-1990	0.0%	0.0%	0.0%	0.0%	0.0%	0.0%	0.0%	0.0%	0.0%	0.0%	0.0%	0.0%	0.0%	0.0%	0.0%	0.0%	0.0%	0.0%	0.0%
1991-2000	0.0%	0.0%	0.0%	0.0%	0.0%	0.0%	0.0%	0.0%	0.0%	0.0%	0.0%	0.0%	0.0%	0.0%	0.0%	0.0%	0.0%	0.0%	0.0%
2001-2010	0.0%	0.0%	0.0%	0.0%	0.0%	0.0%	0.0%	0.0%	0.0%	0.0%	0.0%	0.0%	0.0%	0.0%	0.0%	0.0%	0.0%	0.0%	0.0%
2011-2020	0.1%	0.1%	0.1%	0.2%	0.1%	0.0%	0.0%	0.0%	0.0%	0.0%	0.0%	0.1%	0.0%	0.0%	0.0%	0.0%	0.0%	0.0%	0.0%
after 2020	0.0%	1.0%	0.0%	0.0%	0.0%	0.0%	0.0%	0.0%	0.0%	0.0%	0.0%	0.0%	0.0%	0.0%	0.0%	0.0%	0.0%	0.0%	0.0%

SUPP FIG S6 Analysis of the sequence conservation of the 19 contact (5 Å) residues of compound **22**. For 2,803 RSV A and 2,768 RSV B strain sequences from GISAID, NCBI and Janssen, BLAST sequence alignments were performed. The consensus residues as well as the 2nd and 3rd most frequent amino acids are indicated. Further, the analysis was extended to include the time of origin of the sequences. Both for RSV A and RSV B strains, sequences were grouped in decades of origin. Both absolute counts and percent frequency are reported in the table.

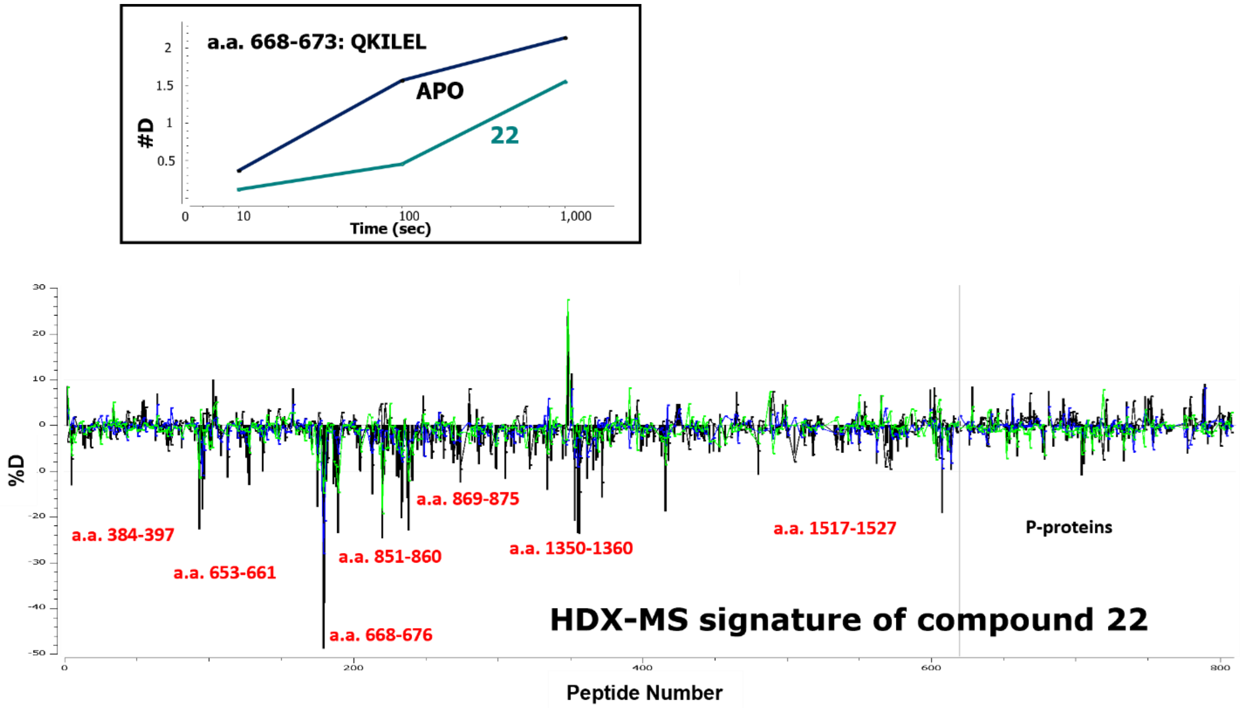

SUPP FIG S7 HDX confirmed the binding epitope of compound **22** and that **22** binding stabilized formation of the helix. Difference in deuterium uptake for RSVpol peptides upon compound **22** engagement plotted for three deuteration timepoints (Black-10s, Blue-100s and Green-1000s). Each bar represents the sum of deuterium uptake for three time points for an individual peptide. Areas with significant change in deuteration are denoted in red. (Inset) Representative deuterium uptake kinetic trace for peptide spanning the stabilized helix (a.a. 668-673) in apo (black) and compound **22**-complex (cyan).

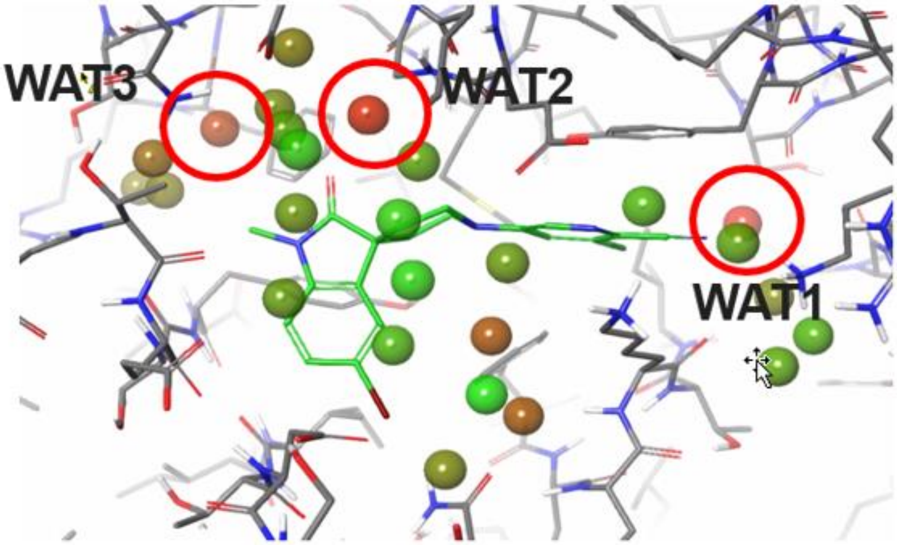

	ΔG	ΔH	-TΔS
WAT1	6.77	3.31	3.46
WAT2	6.01	3.52	2.49
WAT3	5.28	1.70	3.58

SUPP FIG S8 Simulated WaterMap water structure in a 5 Å contact sphere of compound **22**. Water cluster centroids are color-coded from green to red according to increasing excess binding free energy. The inserted table provides excess binding free energy contributions in kcal/mol. Highlighted are water molecules with excess free binding energies larger than +5.0 Kcal/mol.

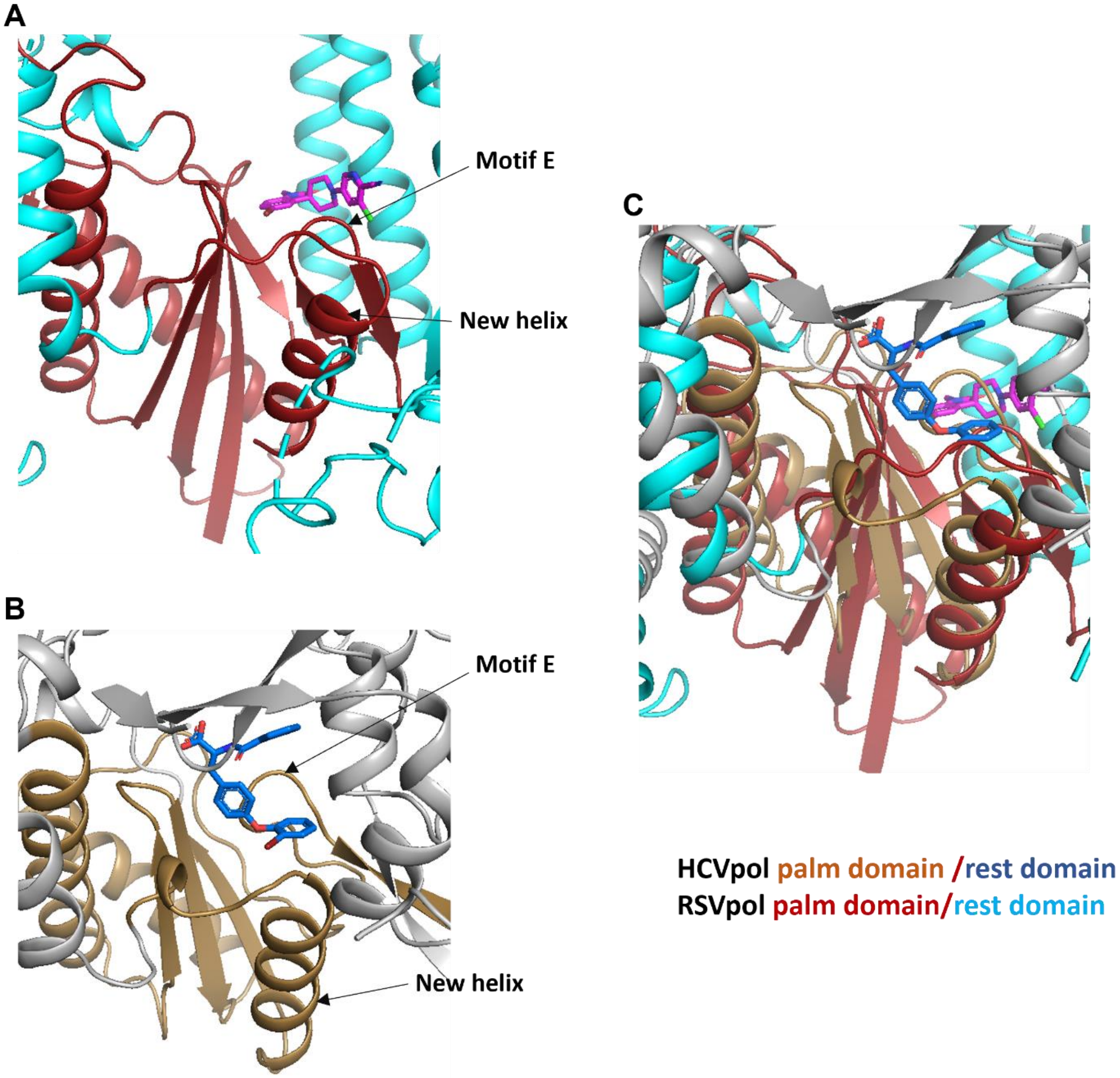

SUPP FIG S9 Structural comparison of inhibitor binding sites in HCV and RSV polymerases. (A) Cartoon depiction of the structure viewed from the binding site, illustrating the 2.72 Å-resolution cryo-EM structure of RSVpol determined in this study. The palm domain is highlighted in red, while the remainder of the RdRp domain is shaded in cyan. (B) Cartoon representation of the binding site of inhibitor PHA-00729145 in the Hepatitis C virus NS5B RNA-dependent RNA polymerase complex (PDB 1YVF). The palm domain is highlighted in orange, while the remainder of the RdRp domain is shaded in gray. (C) Overlay of the binding sites within HCV and RSV polymerase. Despite differences in sequence, both binding sites adopt a similar conformation.

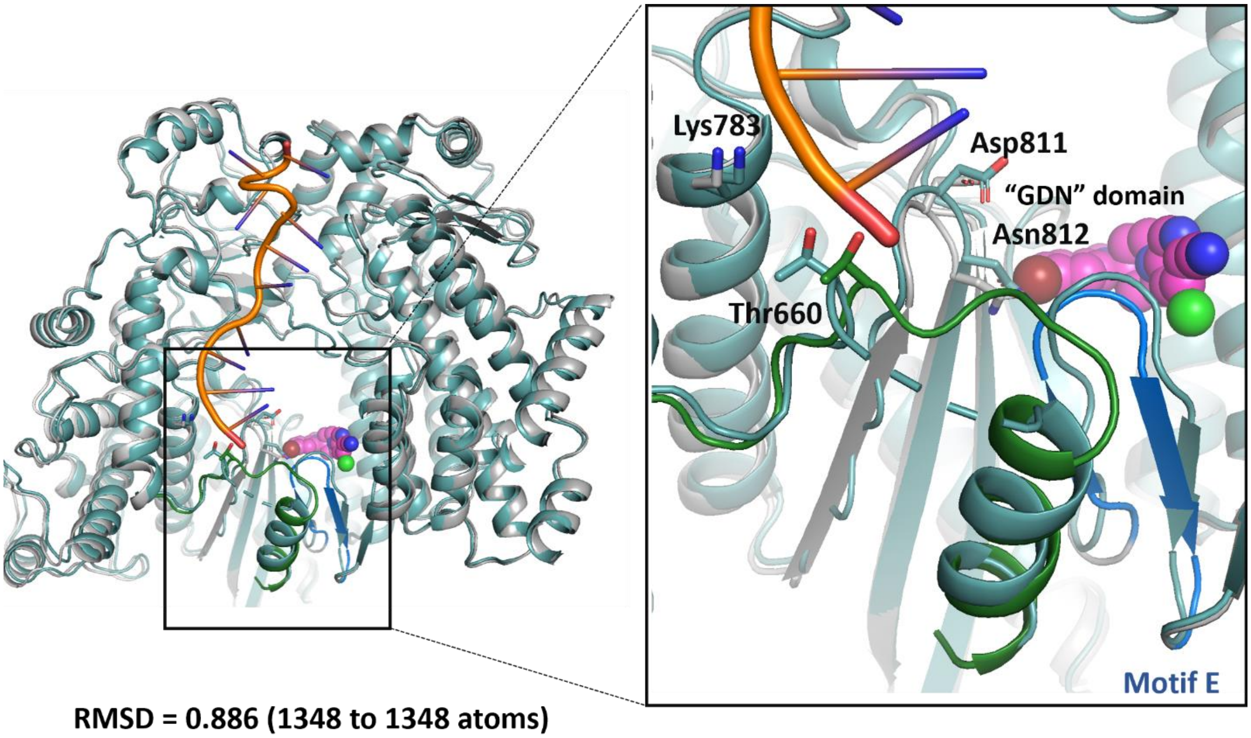

SUPP FIG S10 Structural comparison of compound **22**-bound and Le10-bound RSVpol (L+P) complex (PDB 8SNX). The alignment of RSVpol in complex with Le10 (Le10-bound) and compound **22** is illustrated. The Le10 RNA is depicted in orange, while the protein component is represented in light blue. In the compound **22**-bound RSV structure, Motif E is highlighted in blue, and the supporting helix and loop are indicated in green. Compound **22** is depicted as magenta spheres. The right panels demonstrate the GDN domain and the interaction between key residues Lys783 and Thr660 with RSVpol.

SUPP TABLE S1 Efficacy of compound **22** against clinically isolated RSV A and B strains.

**RSV Strain**	**Compound 22**		
	**EC_{50} (µM)[a]**	**CC_{50} (µM)[b]**	**SI[c]**
RSV-A/Long	0.97	> 50	> 52
RSV-A/A2	1.1	> 50	> 45
RSV-B/Wash/18537	2.7	> 50	> 19
RSV-B/05_036549	1.0	> 50	> 50

[a]Inhibition of viral replication in HeLa cells determined by RT-qPCR, EC_{50} = 50% effective concentration. [b]HeLa cytotoxicity, CC_{50} = 50% cytotoxic concentration. [c]CC_{50}/EC_{50} ratio. Data are representative of two independent experiments.

SUPP TABLE S2 Data collection, reconstruction, and model refinement statistics of cryo-EM structures.

	**RSV L+P + compound 22**<br>**EMD: 48846, PDB: 9N36**
**Data collection and processing**	
Microscope	Glacios
Voltage (keV)	200
Nominal magnification	105000 x
Exposure navigation	Image Shift
Electron exposure (e /Å^2)	40
Total exposure time (sec)	6
Detector	Facon4
Pixel size (Å)*	0.948
Defocus range (μm)	-0.6 to -2.0
Micrographs Used	14890
Final Refined particles (no.)	1546578
**Reconstruction**	
Symmetry imposed	C1
**Resolution**	
FSC threshold 0.143	2.72 Å
**Refinement**	
Protein residues	1643
R.m.s deviations	
Bond lengths (Å)	0.003
Bond angles (°)	0.537
Ramachandran(%)	
Outliers	0.06
Allowed	3.2
Favored	96.74
Rotamer outliers(%)	0.00
MolProbity score	1.45
Clash score	4.75
**Model-to-data fit**	
CC(mask)	0.83
CC(box)	0.69
CC(peaks)	0.69
CC(volume)	0.78

$$\frac{1}{v_{IJ}} = \frac{1}{v_0}\left(1 + \frac{[I]}{K_I} + \frac{[J]}{K_J} + \frac{[I][J]}{\gamma K_I K_J}\right)$$

SUPP EQ 1 Yonetani-Theorell equation (1) where v is the initial reaction velocity, K is the equilibrium dissociation constant, and γ is the interaction constant between two inhibitors, I and J.

General Information

Reagents and solvents were obtained from commercial sources and used as received unless stated otherwise. Flash chromatography purifications were performed on Biotage prepacked silica gel columns using Biotage Isolera or SP4 instruments. The purity of all compounds screened in biological assays was > 95% by HPLC.

NMR analysis

^{1}H NMR spectra were recorded on a Bruker Avance DRX 400 spectrometer or Bruker Avance III 400 spectrometer. NMR spectra were recorded at ambient temperature unless otherwise stated. Data are reported as follow: chemical shift in parts per million (ppm) relative to TMS (δ = 0 ppm) on the scale, integration, multiplicity (s = singlet, d = doublet, t = triplet, q = quartet, quin = quintet, sex = sextet, m = multiplet, br = broad, or a combination of these), coupling constant(s) *J* in Hertz (Hz). Solvents used for the NMR experiments are reported per compound.

HPLC and LCMS

The High Performance Liquid Chromatography (HPLC) measurement was performed using a LC pump, a diode-array (DAD) or a UV detector and a column as specified in the respective methods. If necessary, additional detectors were included (see table of methods below). Flow from the column was brought to the Mass Spectrometer (MS) which was configured with an atmospheric pressure ion source. It is within the knowledge of the skilled person to set the tune parameters (e.g. scanning range, dwell time...) in order to obtain ions allowing the identification of the compound's nominal monoisotopic molecular weight (MW). Data acquisition was performed with appropriate software. Compounds are described by their experimental retention times (Rt) and ions. For molecules with multiple isotopic patterns (Br, Cl), the reported value is the one obtained for the lowest isotope mass. All results were obtained with experimental uncertainties that are commonly associated with the method used. Hereinafter, "SQD" means Single Quadrupole Detector, "BEH" bridged ethylsiloxane/silica hybrid, "DAD" Diode Array Detector.

LCMS Method Codes (Flow expressed in mL/min; column temperature (Col T) in °C; Run time in minutes):

Method code	Instrument	Column	Mobile phase	Gradient	Flow<br>-------<br>Col T	Run time
A	Waters: Acquity® UPLC® - DAD and SQD	Waters :BEH (1.8 µm, 2.1*100 mm)	A: 0.1% NH_4HCO_3 in 95% H_2O + 5% CH_3CN<br>B: CH_3CN	From 100% A to 5% A in 2.10 min, to 0% A in 0.9 min, to 5% A in 0.50 min	0.6<br>-------<br>55	3.5

B	Waters: Acquity® UPLC® - DAD and SQD	Waters :BEH (1.7 µm, 2.1*100 mm)	A: 10mM CH_3COONH_4 in 95% H_2O + 5% CH_3CN B: MeOH	From 95% A to 5% A in 1.3 min, held for 0.7 min	0.8 ------- 55	2
C	Waters: Acquity® UPLC® - DAD and SQD	Waters :BEH (1.7 µm, 2.1*100 mm)	A: 0.1% NH_4HCO_3 in 95% H_2O + 5% CH_3CN B: CH_3CN	From 100% A to 5% A in 2.10 min, to 0% A in 0.9 min, to 5% A in 0.50 min	0.6 ------- 55	3.5
D	Waters: Acquity® UPLC® - DAD and SQD	Waters :BEH (1.8 µm, 2.1*100 mm)	A: 0.1% NH_4HCO_3 in 95% H_2O + 5% CH_3CN B: MeOH	From 100% A to 5% A in 2.10 min, to 0% A in 0.90 min, to 5% A in 0.50 min	0.6 ------- 55	3.5
E	Waters: Acquity® UPLC® - DAD and SQD	Waters :BEH (1.8 µm, 2.1*50 mm)	A: 0.1% NH_4HCO_3 in 95% H_2O + 5% CH_3CN B: CH_3CN	From 100% A to 5% A in 1.3 min, hold 0.70 min	0.8 ------- 55	2
F	Waters: Acquity® UPLC® - DAD and SQD	Waters :BEH (1.8 µm, 2.1*50 mm)	A: 0.1% NH_4HCO_3 in 95% H_2O + 5% CH_3CN B: CH_3CN	From 100% A to 5% A in 1.3 min, hold 0.70 min	0.8 ------- 55	2.0
G	Waters: Acquity® UPLC® - DAD and SQD2	Waters :BEH (1.7 µm, 2.1*50 mm)	A: 10mM CH3COONH4 in 95% H_2O + 5% CH_3CN B: CH_3CN	From 100% A to 5% A in 1.3 min, hold 0.70 min	0.8 ------- 55	2
H	Waters: Acquity® UPLC® - DAD and SQD2	Waters :BEH (1.8 µm, 2.1*100 mm)	A: 0.1% NH_4HCO_3 in 95% H_2O + 5% CH_3CN B: CH_3CN	From 100% A to 5% A in 2.10 min, to 0% A in 0.9 min to 5% A in 0.50 min	0.6 ------- 55	3.5
I	Waters: Acquity® UPLC® - DAD and SQD	Waters :BEH (1.8 µm, 2.1*100 mm)	A: 10mM CH_3COONH_4 in 95% H_2O + 5% CH_3CN B: CH_3CN	From 100% A to 5% A in 2.10 min, to 0% A in 0.90 min, to 5% A in 0.50 min	0.6 ------- 55	3.5
J	Waters: Acquity® UPLC® - DAD and SQD2	Waters :BEH (1.7 µm, 2.1*100 mm)	A: 10 mM NH_4HCO_3 in 95% H_2O + 5% CH_3CN B: MeOH	From 100% A to 5% A in 2.10 min, to 0% A in 0.90 min, to 5% A in 0.50 min	0.6 ------- 55	3.5

HRMS

The liquid chromatography (LC) experiments for the high-resolution mass spectrometry (HRMS) determinations were performed using an Ultimate 3000 RS UHPLC system (Thermo Fisher Scientific, Germering, Germany) composed of a gradient pump, an autosampler, a column oven, and a DAD. A DAD scanning wavelength ranging from 210 to 400 nm was used. Mobile phase A consisted of 0.1% NH_4HCO_3 in 95% H_2O + 5% CH_3CN, and mobile phase B consisted of CH_3CN. The LC experiments were carried out at a flow rate of 0.6 mL/min and were conducted applying a linear gradient from 95% A to 5% A in 2.10 min and held for 1.9 min. The column compartment was kept at 55 °C. A 2.1 mm i.d. × 100 mm Acquity® UPLC BEH C18 column packed with 1.7 um particles was obtained from Waters Corporation (Milford, MA, USA). A 1:10 flow split from the column to the MS spectrometer was applied.

The HRMS experiments were performed in Full MS scan type mode on a Q-Exactive mass spectrometer (Thermo Fisher Scientific, Bremen, Germany) via an electrospray ionization (ESI) interface. The MS was calibrated according to the manufacturer instructions. Nitrogen was used as the nebulizer gas. The MS was operated both in positive and negative mode, and the ESI parameters were as follows: spray voltage: 4.00 kV; capillary temperature: 320 °C; S-lens RF level: 50.0. Masses m/z ranging 100 to 1200 were selected and the experiments were performed at a resolution of 70,000. Xcalibur (version 4.4, Thermo Fisher Scientific) was used as data acquisition software. The reported accurate masses correspond to the $[M+H]^+$ (protonated monoisotopic molecular mass).

Abbreviations

2-Me-THF	2-Methyltetrahydrofuran
ACE-Cl	1-chloroethyl chloroformate
CH_3CN	Acetonitrile
DCM	Dichloromethane
DIAD	Diisopropyl azodicarboxylate
DIPEA	*N*,*N*-Diisopropylethylamine
DMEDA	*N*,*N*-Dimethylethylenediamine
DMF	*N*,*N*-Dimethylformamide
DMSO	Dimethyl sulfoxide
Eq.	equivalents
Et_3N	Triethylamine

EtOAc	Ethyl acetate
EtOH	Ethanol
h	Hour
HPLC	High Performance Liquid Chromatography
HRMS	high-resolution mass spectrometry
i-PrNH$_2$	Isopropylamine
i-PrOH	Isopropyl alcohol
LCMS	Liquid Chromatography Mass Spectrometry
MeOH	Methanol
min	Minute
NaOTMS	Sodium trimethylsilanolate
NMP	N-Methyl-2-pyrrolidone
NMR	Nuclear Magnetic Resonance
Pd(dppf)Cl$_2$	[1,1′-Bis(diphenylphosphino)ferrocene]dichloropalladium(II)<br>CAS [72287-26-4]
Rt	Retention time
RuPhos Pd G4	[Dicyclohexyl(2′,6′-diisopropoxy-2-biphenylyl)phosphine-κP](methanesulfonatato-κO)[2′-(methylamino-κN)-2-biphenylyl-κC2]palladium;<br>CAS [1599466-85-9]
SFC	Supercritical Fluid Chromatography
TFA	Trifluoroacetic acid
THF	Tetrahydrofuran

Synthesis

General procedure A:

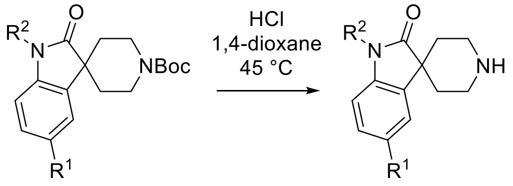

HCl (4 M in dioxane, 3.9 mL, 16 mmol, 5.0 eq.) was added to a mixture of *tert*-butyl 2-oxospiro[indoline-3,4'-piperidine]-1'-carboxylate (3.1 mmol, 1.0 eq.) in 1,4-dioxane (10 mL, 0.3 M) and the mixture was heated to 45 °C and stirred at this temperature for 4 hours. The mixture was allowed to cool to room temperature and the solvent concentrated *in vacuo*. The crude residue was diluted with EtOH (2.0 mL) and concentrated *in vacuo*. The crude product was used in subsequent chemistry without further purification.

General procedure B:

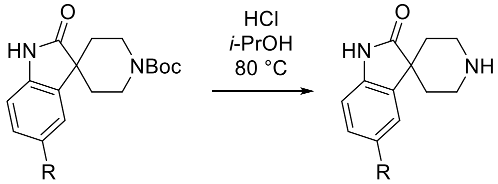

HCl (6 M in *i*-PrOH, 3.8 mL, 23 mmol, 15 eq.) was added to a mixture of *tert*-butyl 2-oxospiro[indoline-3,4'-piperidine]-1'-carboxylate (1.5 mmol, 1.0 eq.) in *i*-PrOH (10 mL, 0.15 M) and the mixture was heated to 80 °C and stirred at this temperature for 30 minutes.
The mixture was allowed to cool to room temperature and the solvent concentrated *in vacuo*. The crude residue was suspended in CH_3CN (5 mL) and filtered. The cake obtained was dried *in vacuo* at 50 °C to afford the desired compound.

General procedure C:

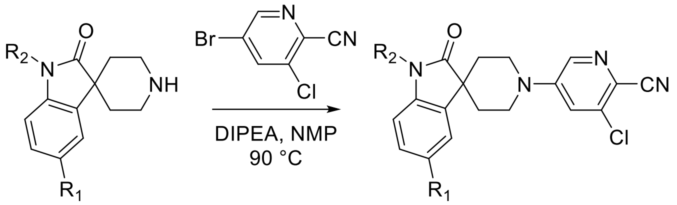

To a vial was charged spiro[indoline-3,4'-piperidin]-2-one (0.60 mmol, 1.0 eq.), 5-bromo-3-chloropyridine-2-carbonitrile (140 mg, 0.60 mmol, 1.0 eq.), DIPEA (0.24 mL, 1.5 mmol, 2.1 eq.) and NMP (2.0 mL, 0.3 M). The mixture was heated at 90 °C for 16 hours. The mixture was allowed to cool to room temperature and poured into water (10 mL). The suspension was filtered to yield the desired compound. Where specified, subsequent purification steps using column chromatography, or trituration, or recrystallization, or Prep HPLC were carried out to obtain the desired compound at > 95% purity by HPLC.

General procedure D:

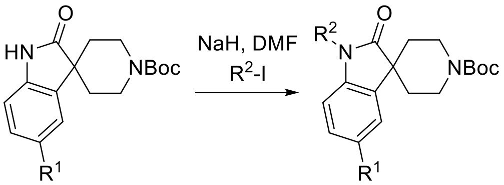

To a stirred solution of *tert*-butyl 2-oxospiro[indoline-3,4'-piperidine]-1'-carboxylate (0.17 mmol, 1.0 eq.) in DMF (1.0 mL, 0.2 M) was added NaH (60% dispersion in mineral oil, 8.6 mg, 0.21 mmol, 1.2 eq.) under a nitrogen atmosphere. The mixture was stirred at room temperature for 1 hour. Alkyl iodide (0.19 mmol, 1.1 eq.) was added and the reaction was further stirred for 1 hour at room temperature. The mixture was diluted with ice water (3.0 mL), allowed to warm to room temperature, and stirred for 16 hours. The suspension was filtered, and the cake obtained was dried *in vacuo* at 45 °C to afford the desired compound.

General procedure E:

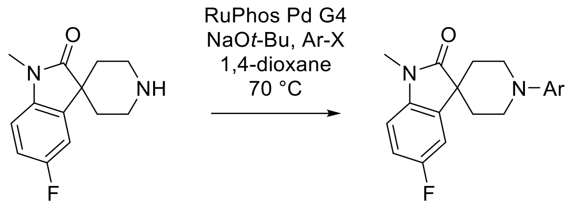

To a vial was charged 5-fluoro-1-methylspiro[indoline-3,4'-piperidin]-2-one (100 mg, 0.37 mmol, 1.0 eq.) and aryl halide (0.56 mmol, 1.5 eq.) in anhydrous 1,4-dioxane (2.0 mL, 0.19 M). The mixture was purged with nitrogen gas for 5 minutes. Base (1.1 mmol, 3.0 eq.) and RuPhos Pd G4 (0.037 mmol, 0.10 eq.) were then added, and the vial was sealed. The mixture was heated at 70 °C for 16 hours. The mixture was allowed to cool to room temperature and concentrated *in vacuo*. The residue was treated with saturated aqueous NH_4Cl solution (5 mL) and extracted with DCM (2 × 5 mL). The organic layer was dried ($MgSO_4$), filtered and concentrated *in vacuo*. The residue was purified using column chromatography or Prep HPLC to afford the desired compound.

Compound 1. (5-(5-bromo-2-oxospiro[indoline-3,4'-piperidin]-1'-yl)-3-chloropicolinonitrile)

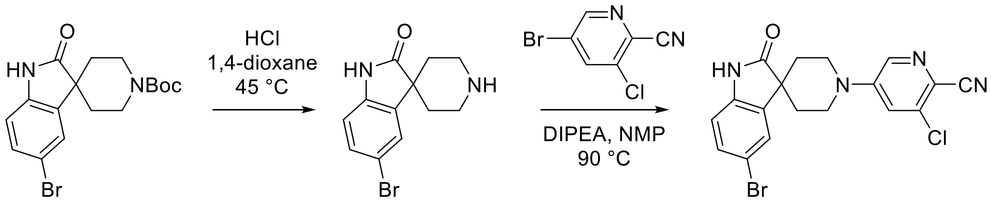

2 9 1

General procedure **A** was followed using with **2** (2.01 g, 5.3 mmol), HCl (4 M in dioxane, 10 mL, 40 mmol) and 1,4-dioxane (10 mL) to afford **9** (1.47 g, 99% yield) as a pale yellow solid. General procedure **C** was then followed, using **9** (1.47 g, 5.2 mmol), 5-bromo-3-chloropyridine-2-carbonitrile (1.13 g, 5.2 mmol), DIPEA (5.1 mL, 31.4 mmol) and NMP (15 mL). After filtration, subsequent purification was performed. The cake obtained was suspended in MeOH (5.0 mL), stirred at room temperature for 30 minutes and filtered. The cake was then dried *in vacuo* at 50 °C for 16 hours to afford the desired compound **1** (1.50 g, 63% yield) as an off-white solid.

Compound 16. (3-chloro-5-(5-chloro-2-oxospiro[indoline-3,4'-piperidin]-1'-yl)picolinonitrile)

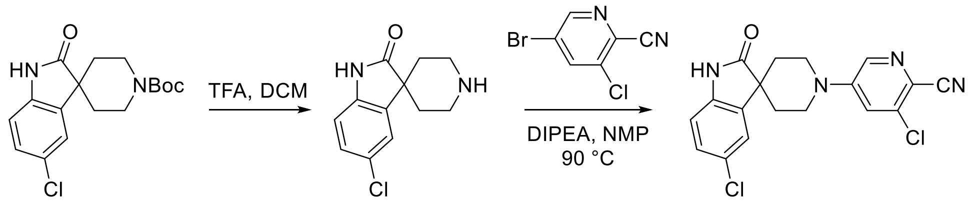

3 10 16

To a solution of **3** (1.00 g, 3.0 mmol) in DCM (25 mL) was added TFA (2.5 mL, 33 mmol) dropwise. The reaction was stirred at room temperature for 48 hours. The reaction mixture was concentrated *in vacuo*. The residue was diluted with water (3 mL) and treated with saturated aqueous $NaHCO_3$ solution until pH > 9. The aqueous layer was extracted with DCM (2 × 3.0 mL).

The organic layer was dried ($MgSO_4$), filtered, and concentrated *in vacuo* to afford **10** (699 mg, 99% yield) as a light yellow paste. General procedure **C** was followed using **10** (0.70 g, 3.0 mmol), 5-bromo-3-chloropyridine-2-carbonitrile (0.71 g, 3.3 mmol), DIPEA (1.1 mL, 6.6 mmol) and NMP (5.0 mL). After filtration, subsequent purification was performed. The cake obtained was recrystallized in CH_3CN/ethanol (9:1 v/v). The suspension was filtered, and the cake obtained was dried *in vacuo* at 45 °C to afford the desired compound **16** (409 mg, 37% yield) as a beige solid.

Compound 17. (3-chloro-5-(5-methyl-2-oxospiro[indoline-3,4'-piperidin]-1'-yl)picolinonitrile)

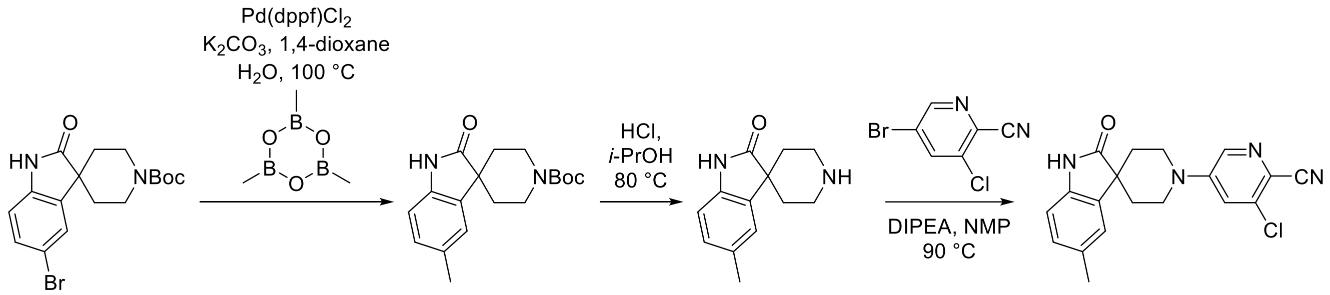

A mixture of **2** (1.00 g, 2.6 mmol), trimethylboroxine (1.1 mL, 7.9 mmol), K_2CO_3 (1.09 g, 7.9 mmol) in 1,4-dioxane (20 mL) and water (5.0 mL) was purged with nitrogen gas for 5 minutes. To the reaction mixture was charged Pd(dppf)Cl_2 (192 mg, 0.26 mmol). The resulting mixture was heated at 100 °C for 16 hours. The mixture was allowed to cool to room temperature, and filtered over Celite, eluting with EtOAc (20 mL). The organic layer was separated, dried ($MgSO_4$), filtered and concentrated *in vacuo*. The crude residue was purified using column chromatography (Heptane/EtOAc gradient 100:0 to 50:50 v/v) to afford **4** (450 mg, 54% yield) as a yellow solid. General procedure **B** was then followed using **4** (450 mg, 1.4 mmol) HCl (6 N in *i*-PrOH, 4.0 mL, 24 mmol) and *i*-PrOH (10 mL). The mixture was allowed to cool to room temperature and the solvent concentrated *in vacuo*. The crude residue was suspended in acetone (5.0 mL) and filtered. The cake was dried *in vacuo* at 50 °C to afford **11** (350 mg, 87% yield) as a white solid. General procedure **C** was then followed using **11** (250 mg, 1.0 mmol), 5-bromo-3-chloropyridine-2-carbonitrile (237 mg, 1.1 mmol), DIPEA (400 µL, 2.5 mmol) and NMP (2.0 mL). After filtration, subsequent purifications were performed. The cake obtained was purified using column chromatography (DCM/MeOH/NH_3 gradient 100:0:0 to 90:9:1 v/v/v) followed by recrystallization in MeOH to afford the desired compound **17** (80.0 mg, 23% yield) as a white solid.

Compound 18. (3-chloro-5-(5-methoxy-2-oxospiro[indoline-3,4'-piperidin]-1'-yl)picolinonitrile)

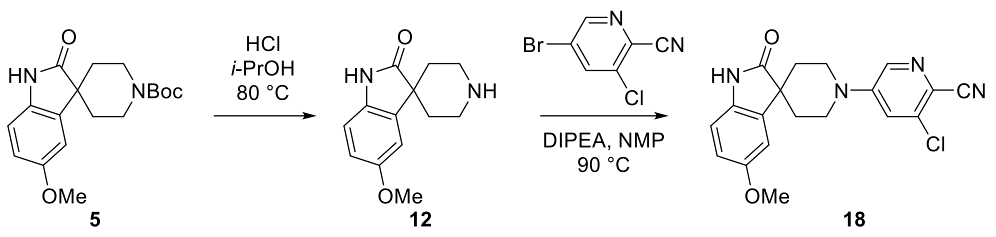

General procedure **B** was followed using **5** (500 mg, 1.5 mmol), HCl (6 M in *i*-PrOH, 3.8 mL, 22.6 mmol) and *i*-PrOH (10 mL) to afford **12** (366 mg, 91% yield) as an off-white solid. General procedure **C** was then followed using **12** (200 mg, 0.74 mmol), 5-bromo-3-chloropyridine-2-carbonitrile (178 mg, 0.82 mmol), DIPEA (0.7 mL, 4.6 mmol) and NMP (1.5 mL). Reaction time was 2 hours. After filtration, subsequent purifications were performed. The cake obtained was purified using column chromatography (Heptane/EtOAc gradient 100:0:0 to 100:0 v/v), followed by trituration with CH_3CN (5.0 mL) to afford the desired compound **18** (100 mg, 36% yield) as a pale-yellow solid.

Compound 19. (3-chloro-5-(5-fluoro-2-oxospiro[indoline-3,4'-piperidin]-1'-yl)picolinonitrile)

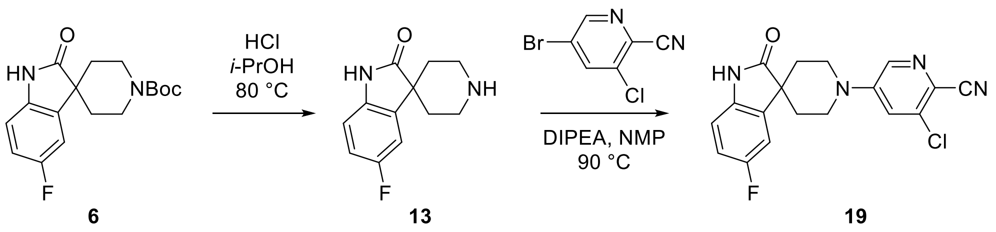

General procedure **B** was followed using **6** (474 mg, 1.4 mmol), HCl (6 M in *i*-PrOH, 2.3 mL, 14 mmol), and *i*-PrOH (10 mL) to afford **13** (320 mg, 89% yield) as a white solid. General procedure **C** was then followed using **13** (157 mg, 0.61 mmol), 5-bromo-3-chloropyridine-2-carbonitrile (133 mg, 0.61 mmol), DIPEA (0.60 mL, 3.8 mmol) and NMP (1.2 mL). The cake obtained after filtration was dried *in vacuo* at 45 °C to afford the desired compound **19** (234 mg, 94% yield) as a pale-yellow solid.

Compound 20. (2-chloro-5-(2-oxospiro[indoline-3,4'-piperidin]-1'-yl)nicotinonitrile)

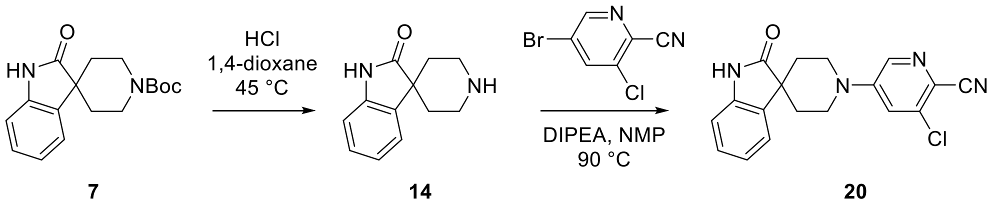

General procedure **A** was followed using **7** (316 mg, 1.0 mmol) and HCl (4 M in dioxane, 2.5 mL, 10 mmol) in 1,4-dioxane (2.1 mL). The reaction was stirred at 100 °C for 20 minutes to afford **14** (202 mg, 95% yield) as a light yellow paste. General procedure **C** was then followed using DIPEA (0.50 mL), 5-bromo-3-chloropyridine-2-carbonitrile (109 mg, 0.50 mmol), **14** (101 mg, 0.50 mmol) in NMP (1.0 mL). After filtration, subsequent purification was performed. The cake

obtained was purified using Prep HPLC (Stationary phase: RP XBridge Prep C18 OBD-10 μm, 50 × 150 mm, Mobile phase: 0.25% NH_4HCO_3 solution in water, CH_3CN) to afford the desired compound **20** (53.0 mg, 31% yield) as a white solid.

Compound 21. (3-chloro-5-(5-fluoro-2-oxo-1-(pyridin-3-yl)spiro[indoline-3,4'-piperidin]-1'-yl)picolinonitrile)

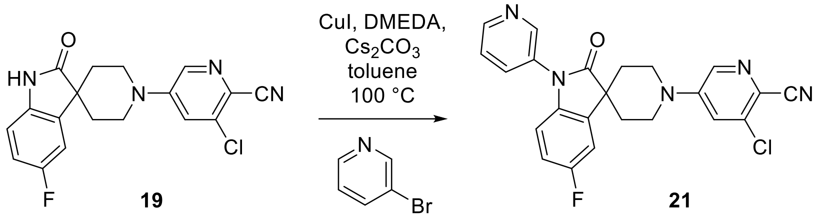

To a microwave vial was charged anhydrous toluene (5.0 mL), CuI (11.4 mg, 0.060 mmol), DMEDA (18 μL, 0.20 mmol), 3-bromopyridine (77 μL, 0.80 mmol), **19** (150 mg, 0.40 mmol) and Cs_2CO_3 (390 mg, 1.2 mmol). The vial was purged with nitrogen gas for 15 minutes and was then capped. The reaction mixture was warmed to 100 °C and stirred at this temperature for 16 hours. The reaction was allowed to cool to room temperature. The reaction was treated with aqueous NH_3 (1.0 mL), stirred for 15 minutes, and further diluted with water (5.0 mL) and DCM (5.0 mL). The mixture was extracted with DCM (3 × 2.0 mL). The combined organic layer was washed with water (5.0 mL), brine (5.0 mL), dried ($MgSO_4$), filtered, and concentrated *in vacuo*. The residue was purified using column chromatography (DCM/MeOH gradient 100:0 to 90:10 v/v) and then Prep HPLC (Stationary phase: RP XBridge Prep C18 OBD-10 μm, 50 × 150 mm, Mobile phase: 0.25% NH_4HCO_3 solution in water, CH_3CN) to afford the desired compound **21** (76.8 mg, 44% yield) as a white solid.

Compound 22. (5-(5-bromo-1-methyl-2-oxospiro[indoline-3,4'-piperidin]-1'-yl)-3-chloropicolinonitrile)

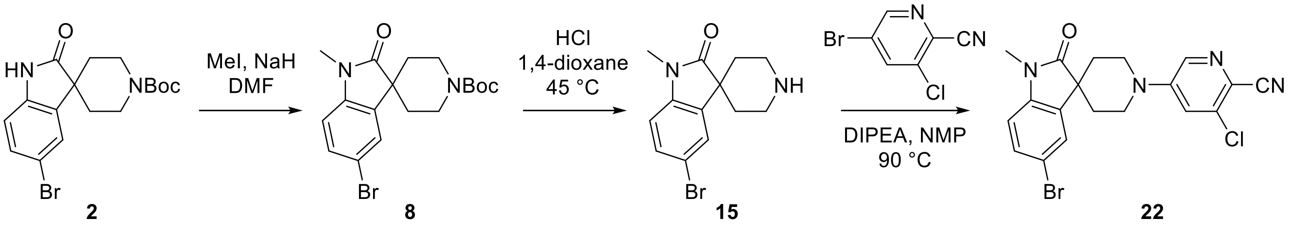

General procedure **D** was followed using NaH 60% in mineral oil (1.15 g, 2.9 mmol), **2** (1.00 g, 2.6 mmol), iodomethane (180 μL, 2.9 mmol) and DMF (10 mL) to afford **8** (1.04 g, 99% yield) as a white solid. General procedure **A** was then followed using **8** (4.50 g, 11 mmol), HCl (4 M in dioxane, 14.2 mL, 57 mmol) and 1,4-dioxane (30 mL) to afford **15** (3.30 g, 88% yield) as an off-white solid. General procedure **C** was then followed using intermediate **15** (2.00 g, 6.0 mmol), DIPEA (3.2 mL, 20 mmol), 5-bromo-3-chloropyridine-2-carbonitrile (1.45 g, 6.7 mmol) in NMP (15 mL). After filtration, subsequent purification was performed. The cake obtained was purified

using column chromatography (Heptane/EtOAc gradient 100:0 to 50:50 v/v) to afford the desired compound **22** (2.05 g, 79% yield) as a light brown solid.

Compound 23. (3-chloro-5-(5-fluoro-1-methyl-2-oxospiro[indoline-3,4'-piperidin]-1'-yl)picolinonitrile)

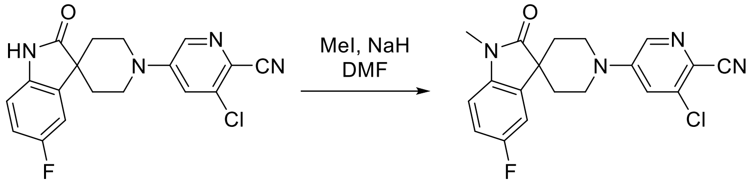

19 23

General procedure **D** was followed using **19** (64.0 mg, 0.17 mmol), NaH (60% dispersion in mineral oil, 8.56 mg, 0.21 mmol), iodomethane (12.0 µL, 0.19 mmol) and DMF (1.0 mL) to afford the desired compound **23** (62.0 mg, 96% yield) as a light yellow solid.

Compound 24. (3-chloro-5-(1-(cyclopropylmethyl)-5-fluoro-2-oxospiro[indoline-3,4'-piperidin]-1'-yl)picolinonitrile)

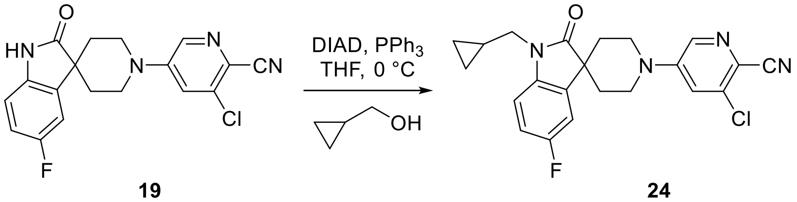

19 24

Under a nitrogen atmosphere, DIAD (129 µL, 0.66 mmol) was added dropwise to a solution of cyclopropane methanol (42 µL, 0.52 mmol), **19** (150 mg, 0.40 mmol) and tributylphosphine (0.16 mL, 0.66 mmol) in THF (2.5 mL) at 0 °C. The reaction mixture was allowed to warm to room temperature and stirred at this temperature for 20 hours. Extra cyclopropane methanol (9.7 µL, 0.12 mmol), DIAD (16 µL, 0.080 mmol) and tributylphosphine (20 µL, 0.080 mmol) were added to the reaction at 0 °C, and the resulting mixture was allowed to warm to room temperature and stirred for 16 hours. The reaction was diluted with water (5.0 mL). The layers were separated and the aqueous layer was extracted with EtOAc (3 × 5.0 mL). The organic layer was washed with water (5.0 mL), brine (5.0 mL), dried ($MgSO_4$), filtered, and concentrated *in vacuo*. The crude residue was purified using column chromatography (Heptane/EtOAc gradient 100:0 to 50:50 v/v) to afford impure product, which was further purified using Prep SFC (Stationary phase: Chiralcel Diacel OD 20 × 250 mm, Mobile phase: CO_2, EtOH + 0.4 *i*-PrNH$_2$) to afford the desired compound **24** (42.0 mg, 26% yield) as a white solid.

Compound 25. (3-chloro-5-(5-fluoro-1-(2-fluoroethyl)-2-oxospiro[indoline-3,4'-piperidin]-1'-yl)picolinonitrile)

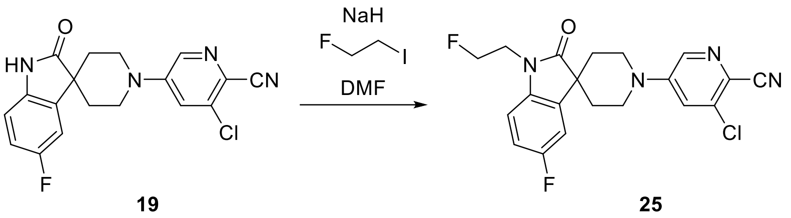

General procedure **D** was followed using **19** (150 mg, 0.30 mmol), NaH (60% dispersion in mineral oil, 18.9 mg, 0.50 mmol), 1-fluoro-2-iodoethane (38 µL, 0.50 mmol) and DMF (1.0 mL). After filtration, the obtained cake was further purified using Prep HPLC (Stationary phase: RP XBridge Prep C18 OBD-10 µm, 30 × 150 mm, Mobile phase: 0.25% NH_4HCO_3 solution in water, MeOH) to afford the desired compound **25** (62.5 mg, 40% yield) as a light yellow solid.

Compound 29. (2-chloro-6-(5-fluoro-1-methyl-2-oxospiro[indoline-3,4'-piperidin]-1'-yl)nicotinonitrile)

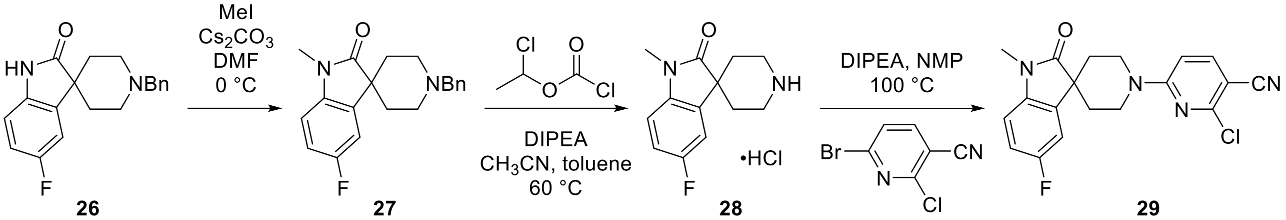

To a solution of **26** (13.0 g, 42 mmol) in DMF (250 mL) was added Cs_2CO_3 (41.0 g, 126 mmol). The mixture was stirred at room temperature for 1 hour. The mixture was cooled to 0 °C and a solution of methyliodide (2.9 mL, 46 mmol) in DMF (20 mL) was added over 35 minutes at 0 °C. The resulting mixture was stirred at this temperature for further 4 hours. LCMS showed 62% conversion to the desired product and 3% quaternization product. A solution of methyliodide (1.1 mL, 17 mmol) in DMF (10 mL) was added over 25 minutes to the reaction at 0 °C. LCMS showed 12% quaternization product. The mixture was poured onto ice water (1.0 L) and allowed to warm to room temperature and stirred for 1 hour. The suspension was filtered, and the cake obtained was dried *in vacuo* at 50 °C for 16 hours. The aqueous filtrate was extracted with EtOAc (2 × 100 mL). The combined organic layer was washed with brine (100 mL), dried ($MgSO_4$), filtered, and concentrated *in vacuo*. This residue and the dried cake were combined to afford **27** (10.2 g, 75% yield) as a brown solid.

1-chloroethyl chloroformate (4.6 mL, 42 mmol) was added dropwise to a solution of **27** (9.50 g, 29 mmol) and DIPEA (1.4 mL, 8.4 mmol) in CH_3CN:toluene (1:1, v/v, 280 mL) at 60 °C. The reaction was stirred at 60 °C for 1 hour. The reaction mixture was allowed to cool to room temperature and concentrated *in vacuo*. The residue was dissolved in MeOH (10 mL), stirred at 50 °C for 1 hour, allowed to cool to room temperature, and the solvent concentrated *in vacuo*. The residue was suspended in diisopropylether (5.0 mL) and CH_3CN (0.1 mL), treated with HCl solution (6 N in *i*-PrOH) until pH < 2, and stirred

at room temperature for 1 hour. The suspension was filtered, and the cake obtained was dried *in vacuo* at 50 °C to afford the HCl salt of **28** (5.10 g, 65% yield) as a white solid.

General procedure **C** was followed using 6-bromo-2-chloronicotinonitrile (80.3 mg, 0.4 mmol), **28** (100 mg, 0.4 mmol) and DIPEA (0.36 mL, 2.2 mmol) in NMP (1.1 mL). The reaction was stirred at 100 °C for 1 hour to afford the desired compound **29** (104 mg, 76% yield) as a white solid.

Compound 30. (4-(5-fluoro-1-methyl-2-oxospiro[indoline-3,4'-piperidin]-1'-yl)-2-methylbenzonitrile)

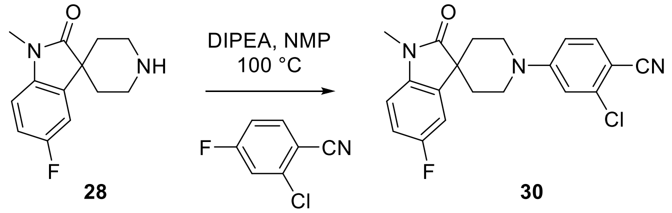

To a vial was charged 2-chloro-4-fluoronitrile (77.9 mg, 0.50 mmol), **28** (80.0 mg, 0.30 mmol), DIPEA (0.19 mL, 1.2 mmol) and NMP (1.0 mL). The reaction mixture was heated at 100 °C for 16 hours. The reaction was allowed to cool to room temperature and was diluted with ice water (5.0 mL). The mixture was allowed to warm to room temperature and extracted with EtOAc (3 × 3.0 mL). The organic layer was dried ($MgSO_4$) and concentrated *in vacuo*. The residue was purified using column chromatography (DCM/MeOH gradient 100:0 to 95:5 v/v) to afford the desired compound **30** (68.0 mg, 63% yield) as a yellow solid.

Compound 31. (5-(5-fluoro-1-methyl-2-oxospiro[indoline-3,4'-piperidin]-1'-yl)-3-methoxypicolinonitrile)

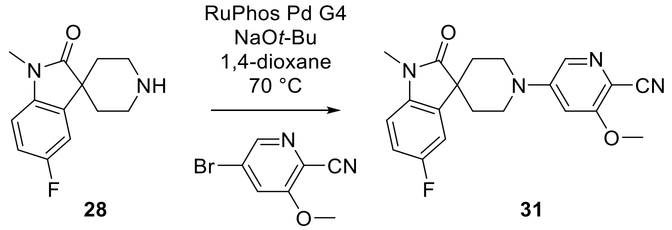

General procedure **E** was followed using **28** (100 mg, 0.37 mmol), 5-bromo-3-methoxypicolinonitrile (118 mg, 0.56 mmol), NaO*t*-Bu (2 M in THF, 0.55 mL, 1.1 mmol), and RuPhos Pd G4 (31.0 mg, 0.037 mmol) in anhydrous 1,4-dioxane (2.0 mL). After 16 hours, extra 5-bromo-3-methoxypicolinonitrile (39.3 mg, 0.18 mmol), NaO*t*-Bu (2 M in THF, 0.19 mL, 0.37 mmol) and RuPhos Pd G4 (31.0 mg, 0.037 mmol) were added to the reaction. After work up, a purification was performed by Prep HPLC (Stationary phase: RP XBridge Prep C18 OBD-10 µm, 30 × 150 mm, Mobile phase: MeOH) to afford impure product, which was further purified using

column chromatography (EtOAc/Heptane gradient 0:100 to 50:50 v/v) to afford the desired compound **31** (17.9 mg, 13% yield) as a white solid.

Compound 32. (2-fluoro-4-(5-fluoro-1-methyl-2-oxospiro[indoline-3,4'-piperidin]-1'-yl)-6-methylbenzonitrile)

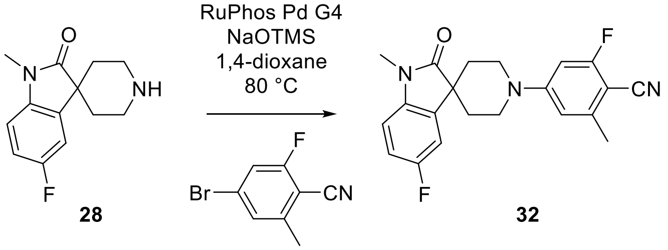

A reaction vial was charged with **28** (27.1 mg, 0.10 mmol), 4-bromo-2-fluoro-6-methylbenzonitrile (42.8 mg, 0.20 mmol), RuPhos Pd G4 (8.5 mg, 0.010 mmol) and NaOTMS (33.7 mg, 0.30 mmol) in anhydrous 1,4-dioxane (2.0 mL). The resulting mixture was stirred at 80 °C for 20 hours. The reaction mixture was allowed to cool to room temperature. SiliaMetS Dimercaptotriazine (50 mg) was added and the reaction mixture was stirred for 2 hours. The resulting mixture was diluted with EtOAc (2.0 mL) and water (1.0 mL), and transferred to a Tecan liquid handler. The layers were separated and the aqueous layer was extracted with EtOAc (2 × 2.0 mL). The organic layer was filtered, diluted with DMSO (0.75 mL) and concentrated *in vacuo*. The residue was diluted with MeOH/CH_3CN (1:1 v/v, 2 mL) and purified using Prep HPLC (Stationary phase: RP XBridge Prep C18 OBD-10 µm, 30 × 150 mm, Mobile phase: 0.25% NH_4HCO_3 solution in water, CH_3CN) to afford the desired compound **32** (15.8 mg, 40% yield) as a light yellow solid.

Compound 34. (1'-(5-chloro-6-morpholinopyridin-3-yl)-5-fluoro-1-methylspiro[indoline-3,4'-piperidin]-2-one)

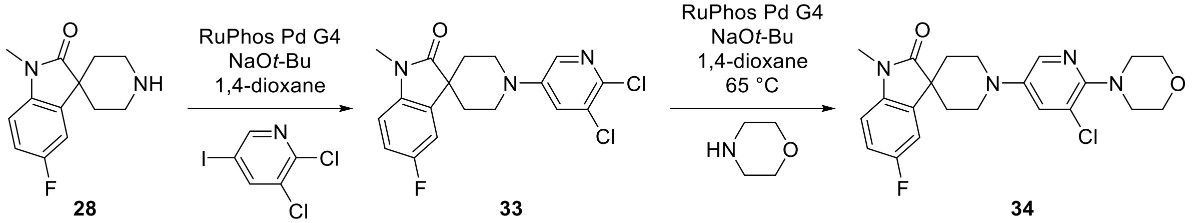

General procedure **E** was followed using **28** (720 mg, 3.1 mmol), 2,3-dichloro-5-iodopyridine (878 mg, 3.2 mmol), RuPhos Pd G4 (261 mg, 0.31 mmol), NaO*t*-Bu (2 M in THF, 4.6 mL, 9.2 mmol) in anhydrous 1,4-dioxane (7.0 mL). The mixture was stirred at room temperature for 16 hours. After work up, the obtained residue was purified using column chromatography (Heptane/EtOAc gradient 100:0 to 50:50 v/v) to afford to afford **33** (840 mg, 68% yield) as a light yellow solid. A solution of **33** (100 mg, 0.26 mmol) in anhydrous 1,4-dioxane (1.0 mL) was purged with nitrogen gas for 15 minutes. To this solution was added morpholine (22.5 µL, 0.26 mmol), NaO*t*-Bu (2 M in THF, 0.39 mL, 0.78 mmol) and RuPhos Pd G4 (11.1 mg, 0.013 mmol). The

reaction mixture was heated to 65 °C and stirred at this temperature for 18 hours. The reaction was allowed to cool to room temperature and treated with saturated aqueous NH_4Cl solution (1.0 mL). The layers were separated and the aqueous layer was extracted with 2-Me-THF (3 × 15 mL). The combined organic layer was washed with brine (15 mL), dried (Na_2SO_4) and concentrated *in vacuo*. The obtained residue was purified using column chromatography (Heptane/EtOAc gradient 100:0 to 50:50 v/v) to afford impure product, which was further purified using Prep HPLC (Stationary phase: RP XBridge Prep C18 OBD-10 µm, 30 × 150 mm, Mobile phase: 0.25% NH_4HCO_3 solution in water, CH_3CN) to afford the desired compound **34** (25.0 mg, 20% yield) as an off-white solid.

1. **Theorell H, Yonetani T.** 1964. Studies on Liver Alcohol Dehydrogenase Complexes. Iv. Spectrophotometric Observations on the Enzyme Complexes. Arch Biochem Biophys **106:**252-258.

2. **Yu X, Abeywickrema P, Bonneux B, Behera I, Anson B, Jacoby E, Fung A, Adhikary S, Bhaumik A, Carbajo RJ, De Bruyn S, Miller R, Patrick A, Pham Q, Piassek M, Verheyen N, Shareef A, Sutto-Ortiz P, Ysebaert N, Van Vlijmen H, Jonckers THM, Herschke F, McLellan JS, Decroly E, Fearns R, Grosse S, Roymans D, Sharma S, Rigaux P, Jin Z.** 2023. Structural and mechanistic insights into the inhibition of respiratory syncytial virus polymerase by a non-nucleoside inhibitor. Commun Biol **6:**1074.
